# Cell-Penetrating Peptides—Mechanisms of Cellular Uptake and Generation of Delivery Systems

**DOI:** 10.3390/ph3040961

**Published:** 2010-03-30

**Authors:** Sara Trabulo, Ana Luísa Cardoso, Miguel Mano, Maria C. Pedroso de Lima

**Affiliations:** 1 Center for Neuroscience and Cell Biology of Coimbra, Department of Zoology, University of Coimbra, Portugal; 2 Department of Life Sciences, Faculty of Science and Technology, University of Coimbra, Apartado 3126, 3001-401 Coimbra, Portugal

**Keywords:** cell-penetrating peptides, nucleic acid delivery, non-viral vectors

## Abstract

The successful clinical application of nucleic acid-based therapeutic strategies has been limited by the poor delivery efficiency achieved by existing vectors. The development of alternative delivery systems for improved biological activity is, therefore, mandatory. Since the seminal observations two decades ago that the Tat protein, and derived peptides, can translocate across biological membranes, cell-penetrating peptides (CPPs) have been considered one of the most promising tools to improve non-invasive cellular delivery of therapeutic molecules. Despite extensive research on the use of CPPs for this purpose, the exact mechanisms underlying their cellular uptake and that of peptide conjugates remain controversial. Over the last years, our research group has been focused on the S4_13_-PV cell-penetrating peptide, a prototype of this class of peptides that results from the combination of 13-amino-acid cell penetrating sequence derived from the Dermaseptin S4 peptide with the SV40 large T antigen nuclear localization signal. By performing an extensive biophysical and biochemical characterization of this peptide and its analogs, we have gained important insights into the mechanisms governing the interaction of CPPs with cells and their translocation across biological membranes. More recently, we have started to explore this peptide for the intracellular delivery of nucleic acids (plasmid DNA, siRNA and oligonucleotides). In this review we discuss the current knowledge of the mechanisms responsible for the cellular uptake of cell-penetrating peptides, including the S4_13_-PV peptide, and the potential of peptide-based formulations to mediate nucleic acid delivery.

## 1. Introduction 

During the last two decades a number of peptides presenting the ability to be translocated across biological membranes were identified and thoroughly studied, resulting in the characterization of a new family of peptides known as cell-penetrating peptides (CPPs), in some cases also frequently referred to as protein transduction domains (PTDs) [1]. The profound interest that CPPs evoked among the scientific community was associated not only with their ability to cross cellular membranes by a non-toxic process, apparently independent of membrane receptors and energy consumption, but mainly due to their capacity to promote the efficient cellular internalization of biomolecules associated to these peptides. Since the lack of permeability of the cellular membranes to hydrophilic biomolecules constitutes one of the most important barriers to the delivery of therapeutic agents, this discovery has been regarded as an important step towards the development of novel strategies to increase the intracellular availability of molecules with high therapeutic interest but low membrane permeability, such as peptides, proteins and nucleic acids. Regardless of the great variability in their amino acid sequence, cell-penetrating peptides are usually short peptide sequences rich in basic amino acids (lysine and arginine), in some cases exhibiting the ability to be arranged in amphipathic alpha-helical structures. Among all CPPs described to date, which include protein transduction domains, chimeric peptides and peptides of synthetic origin, the peptides derived from the HIV-1 Tat protein [2,3] and from the homoeodomain of the Antennapedia protein of Drosophila [4,5] (Tat and Penetratin peptides, respectively), as well as the synthetic Pep-1 peptide [6], are among the best characterized. These peptides have been successfully used for the intracellular delivery of different cargoes [7,8,9,10,11,12,13,14,15,16,17,18,19], including nanoparticles, full-length proteins, liposomes and nucleic acids, both *in vitro* and *in vivo*, resulting in successful transduction in animal tissues, including the brain. 

Despite the extensive use of CPPs for delivery purposes, the exact mechanisms underlying their cellular uptake, and that of peptide conjugates, remain poorly understood and are still the object of some controversy. In contrast with other classes of peptides (such as fusogenic peptides of viral origin and antimicrobial peptides) which are also able to cross cellular membranes, the mechanisms behind CPP internalization are highly efficient and harmless to the cells, avoiding membrane destabilization and loss of cellular integrity. However, the high heterogeneity present in this family of peptides, together with contradicting reports later attributed to cell fixation-derived artifactual observations [20,21,22], have hampered the clarification of the exact mechanisms responsible for CPP uptake. 

In this review we discuss several mechanisms of cellular internalization described for CPPs, in the presence or absence of cargo, with a special emphasis on the S4_13_-PV peptide, a karyophilic CPP [23] which has been extensively studied in our laboratory, both in terms of its cellular uptake, as well as of its potential for delivery of biomolecules. We also discuss recent advances in the use of CPPs, including the S4_13_-PV peptide, for the delivery of DNA and siRNAs, aiming at their application in a therapeutic context.

## 2. Mechanisms of Cellular Internalization of CPPs

Initial reports that CPP internalization occurred even at low temperatures excluded endocytotic pathways as the main mechanism responsible for the uptake of these peptides and suggested the existence of alternative energy-independent internalization mechanisms. Studies employing peptides prepared with D enantiomers and peptides with reverted sequences demonstrated that the translocation efficiency of these peptides was similar (or superior, in the case of D enantiomers) to that of corresponding L enantiomers and non-reversed peptides, also dismissing the involvement of membrane receptors in peptide uptake [24,25,26]. As a result of these observations, several models were proposed to explain CPP translocation across cellular membranes as a consequence of a direct interaction of this class of peptides with phospholipids and other membrane components.

Later, several studies suggested that the apparent membrane translocation of CPPs and their accumulation in intracellular compartments was due to artifacts related to cell fixation rather than to an energy-free and receptor-independent uptake process [20,21,22]. These observations led to a full re-evaluation of the mechanisms involved in CPP internalization. 

Results from comprehensive re-evaluation studies, performed in live cells, provided evidence that in addition to the already described endocytosis-independent mechanisms, involving the direct translocation of CPPs through cellular membranes, several endocytotic pathways, such as caveolae-mediated endocytosis [27,28], macropiynocytosis [29,30] and clathrin-mediated endocytosis [31], played a role in peptide internalization. Furthermore, several differences were observed between the internalization of unconjugated CPPs and CPPs conjugated with high molecular-weight molecules, such as proteins and DNA, suggesting the existence of distinct internalization mechanisms for the same peptide, depending on the presence or absence of cargo. Moreover, and in agreement with the efficient uptake of CPPs by a vast number of cell types and tissues, an important role was attributed to cell surface heparan sulfate proteoglycans (HSPG) in the CPP internalization process. It should be emphasized that proteoglycan contribution to CPP internalization is consistent with any of the possible uptake mechanisms discussed so far [32]. Indeed, biding of these permeating peptides to cell surface proteoglycans could promote the interaction of CPPs with the cellular membranes, facilitating the subsequent interactions necessary to the translocation process; in an alternative scenario, this same binding step could induce by itself certain endocytotic mechanisms, leading to CPP internalization. According to recent studies, the cellular internalization of the R9 peptide and of other arginine oligomers was shown to be mediated by an endocytotic mechanism dependent on peptide binding to heparan sulfate proteoglycans [33]. The authors of this study also proposed that once inside the endosome, the heparan sulfate chains would be degraded by heparanases, leading to dissociation of the peptides and consequent interaction of the CPPs with the endosomal membrane, thus promoting its destabilization and peptide release into the cytoplasm. 

**Figure 1:**
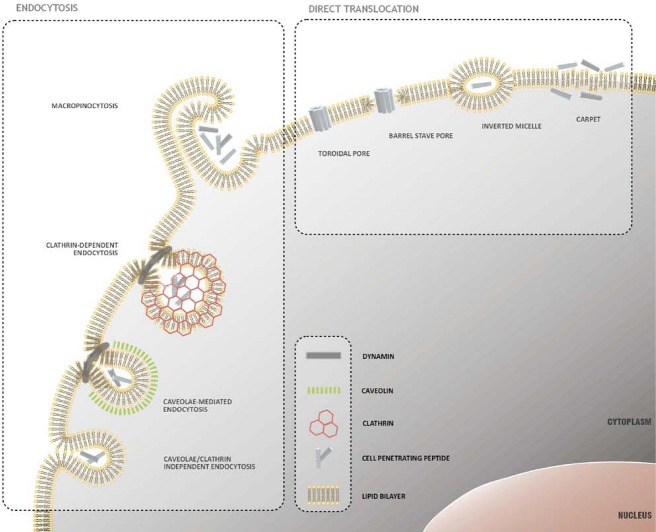
Mechanisms of peptide uptake across the cellular membrane. A variety of internalization mechanisms have been proposed to explain cellular uptake of CPPs. These mechanisms include well-characterized energy-dependent pathways, based on vesicle formation and collectively referred as endocytosis, and direct translocation or cell penetration models, which involve the formation of hydrophilic pores or local destabilization of the lipid bilayer.

Below we illustrate the different mechanisms proposed to explain the internalization of free or cargo-conjugated CPPs (Figure 1). These mechanisms fall into two broad categories: endocytosis and direct membrane translocation. 

### 2.1. Direct Translocation of CPPs across Biological Membranes

The models proposed to explain the direct translocation of CPPs across biological membranes include the “inverted micelle model”, the models involving the formation of membrane pores and the “carpet model”. According to the “inverted micelle model”, described initially by Derossi *et al.* [24] in an attempt to explain the results obtained with the pAntp peptide, the interaction of cell-penetrating peptides with biological membranes would lead to a disturbance of the lipid bilayer, resulting in the formation of inverted hexagonal structures (inverted micelles). The peptides would be trapped in the hydrophilic environment present in the micelle core until their interaction with the membrane components would lead to the occurrence of an inverse process, resulting in the destabilization of the inverted micelles and consequent release of the peptides into the intracellular compartment. This model is supported by data obtained from nuclear magnetic resonance (NMR) studies showing that the interaction of the pAntp peptide with membranes can result in the formation of inverted structures [34]. Additionally, this model provides an acceptable explanation to the translocation of a small hydrophilic peptide across a lipid membrane, without having to overcome the energetic barrier presented by the hydrophobic interior of the lipid bilayer. However, the “inverted micelle model” is not compatible with the translocation of high molecular weight conjugates, since the formation of these inverted hexagonal structures, containing molecules of considerable size in their hydrophilic core, is not likely to occur.

By analogy with the mechanisms of membrane disturbance initially proposed to explain the translocation of antimicrobial peptides and toxins, alternative models were described to explain CPP uptake. According to the models involving the formation of “barrel stave” or toroidal pores, the translocation of peptides and their conjugates across biological membranes would result from the formation of transient pores, produced upon peptide insertion into the membrane, and oligomerization of the inserted peptides in a ring-shape structure. In the case of “barrel stave” pores, the peptides would assume an amphipathic alpha-helix structure when inserted into the membrane, where the hydrophobic face of the amphipathic helices would interact with the aliphatic chains of the bilayer phospholipids and the hydrophilic face would form the interior of the pore [35,36,37,38]. The model involving the formation of toroidal pores is similar, except that in this model the peptides inserted in the membrane would interact exclusively with the polar groups of membrane phospholipids, inducing significant rearrangement of the lipid bilayer [38,39]. According to the “carpet model”, the membrane translocation of permeating peptides and their conjugates would occur as a consequence of a transient destabilization of the cellular membrane, induced by the extensive association of the peptide to its surface, and consequent phospholipid reorganization [35,36,37,38].

Although the previously described models share several common features, it is important to highlight some significant differences: (i) according to the “inverted micelle model”, the peptides remain associated to the membrane surface during translocation and never experience direct contact with the hydrophobic interior of the lipid bilayer, in contrast to what is described in the models that assume pore formation, where the insertion of the peptides in the membrane and the resulting transmembrane conformation are important steps of the translocation process; (ii) both the “toroidal pore” and the “carpet model” describe an extensive reorganization of membrane phospholipids, in contrast to the “barrel stave” model, in which the structure of the lipid bilayer would not be significantly disturbed; (iii) the interaction of cell-penetrating peptides with cellular membranes would result in the formation of concave membrane surfaces according to the “inverted micelle” model, whereas convex membrane surfaces would be formed according to the “toroidal pore” model; (iv) the models involving the formation of “barrel-stave” or “toroidal” pores, in which homo-oligomerization of the membrane-inserted peptides occurs, predicts the existence of a well-defined structure, in contrast with the highly disorganized structure responsible for the destabilization of the cellular membrane described in the “carpet model”. 

With the exception of the “inverted micelle” model, all reported models are compatible with the translocation of large size molecules across biological membranes. In addition, these models require the presence of amphipathic alpha-helix secondary structures, a feature shared by many CPPs. However, the translocation of large molecules by any of these mechanisms would imply an extensive destabilization of the cellular membrane, not compatible with the low cytotoxicity usually associated with the membrane translocation of CPPs and their conjugates. Accordingly, we can conclude that none of the above described models completely explains all the experimental data obtained with different CPPs, indicating that alternative mechanisms should play a role in peptide translocation, specially when conjugated with high molecular weight cargoes. 

### 2.2. Endocytosis as a Pathway for CPP Internalization

Although recent studies have clearly demonstrated the involvement of endocytosis in the internalization of several CPPs and their conjugates, some controversy still exists regarding the exact endocytotic pathways which contribute to this process. 

Endocytosis comprises different cellular mechanisms responsible for the uptake of biomolecules, toxins and even other cells. These mechanisms can be divided into two main categories: phagocytosis, a process which occurs only in specialized cells, such as macrophages, and pinocytosis, a set of internalization pathways active in most cells, which includes macropinocytosis, clathrin-mediated endocytosis, caveolae-mediated endocytosis and other less well-characterized pathways [40,41,42,43]. 

Studies performed to clarify the involvement of these internalization pathways in CPP translocation have employed different experimental approaches such as: (i) peptide/cell interaction at low temperatures (approximately 4ºC) or in energy depletion conditions; (ii) incubation with drugs that selectively compromise different internalization pathways; (iii) evaluation of peptide or peptide conjugate co-localization with molecules known to be internalized by specific endocytotic pathways (e.g., transferrin, cholera toxin G subunit) or with molecular markers of known internalization pathways (e.g., Caveolin-1, early endosome antigen-1 - EEA1); and (iv) overexpression of dominant negative mutants of proteins involved in the internalization process (e.g., Dynamin). By using these approaches, different studies demonstrated the contribution of several pinocytosis pathways to the cellular internalization of Tat. The fusion protein GST-Tat-GFP was found to enter cells mainly by caveolae-mediated endocytosis [28,44], while the Tat peptide and the Tat-HA2 fusion peptide were described to be internalized mainly through macropinocytosis [29,30,45,46]; clathrin-coated vesicles have also been implicated in the internalization of unconjugated Tat peptide [31]. To prevent possible side-effects associated with the use of endocytosis inhibitors Ter-Avetisyan *et al.*, performed studies on the uptake of arginine-rich peptides in genetically engineered cells, lacking functional clathrin-mediated or cavoleae-dependent internalization pathways [47]. In parallel, experiments the authors took advantage of physical methods, such as temperature decrease, to inhibit all endocytotic pathways simultaneously. In this study Tat was not excluded from cells in any of the tested conditions, suggesting that Tat cell uptake can also be endocytosis-independent.

In another interesting work, Duchardt and collegues [42] compared the cellular uptake of three known CPPs: Antennapedia homeodomain-derived peptide (Antp), Tat and the nona-arginine peptide R9. The authors concluded that all three peptides simultaneously use three endocytotic pathways: macropinocytosis, clathrin-mediated endocytosis and caveolae-dependent endocytosis. The Antp peptide was found to differ from the other two peptides in the extent by which the different mechanisms contribute to CPP uptake, showing a higher contribution of clathrin-mediated endocytosis. Moreover, the authors also reported a endocytosis-independent internalization pathway for Antp, present at high peptide concentrations. The differences in the results obtained in all these studies can in part be explained by the unspecificity and toxicity associated with the endocytosis inhibitors frequently employed in this kind of experiments [47,48] and by the different experimental conditions with respect to cell lines, incubation times and peptide concentrations, but can also translate different internalization mechanisms associated with the same peptide.

Regarding cargo-associated CPPs, most studies suggest that endocytosis is the main mechanism responsible for CPP-cargo uptake, although the exact pathway of internalization can vary according to both peptide and cargo properties. For example, Lundin *et al.* [49] compared the internalization route of several cationic and amphipathic CPPs, including penetratin, M918, transportan and a modification of transportan referred as TP10 [50] when conjugated with PNA molecules. The results suggested that while cationic conjugates relied more on macropinocytosis for internalization, amphipathic peptides conjugated with PNAs were internalized mainly by clathrin-mediated endocytosis.

Independently of the endocytotic pathway responsible for the cellular internalization of a certain CPP or its conjugate, several studies have suggested that endosomal release is the main obstacle to the intracellular delivery and biological activity of the conjugated molecules [29,51]. In this context, several strategies have been developed to overcome the endosomal barrier, such as the use of drugs that prevent endosomal acidification [29,52], fusogenic peptides or photosensitizer molecules capable of promoting endosome disruption [53,54]. Chloroquine [29,52] is an example of a well-known endosomolytic reagent, which has been largely employed in combination with CPPs. Several studies have demonstrated an enhancement of the activity of cargo molecules following addition of chloroquine to the cell culture medium. An illustrative example is the work of Veldhoen *et al.* [55], which showed that siRNA delivery by MPGα, a peptide derived from MPG by mutation of its hydrophobic domain, was potentiated in the presence of this drug, leading to an increase in gene silencing efficiency. However, this kind of chemical reagents may not be suitable for *in vivo* therapeutic use, since their effective concentrations are often associated with high cytotoxicity. As an alternative strategy, peptides which promote the destabilization of the endosome membrane upon acidification of this compartment have been employed to facilitate the release of the endosome-entrapped molecules in the absence of significant toxicity. In 2004, Wadia and colleagues described a strategy to enhance the endosomal release of a Tat-Cre construct, in which the Tat peptide was fused to the HA2 pH-sensitive fusogenic peptide derived from the hemagglutinin protein of influenza virus [29]. As expected, the resulting construct had higher transduction efficiency than the initial Tat-Cre peptide and led to a significant enhancement of the biological activity of this CPP [29]. In addition to endosomolytic reagents and fusogenic peptides, photosensitizer and fluorescent molecules have also been used to enhance the diffusion of molecules from endosomes to the cytosol. Maiolo *et al.* [53] demonstrated that fluorescent dyes covalently bound to penetratin and polyarginine peptides led to cytosolic diffusion of these CPPs, following photostimulation at a wavelength close to the excitation maximum of the fluorescent dye. This excitation is proposed to generate reactive oxygen species and induce rupture of the endosomal membrane. In another study [56] a similar photostimulation strategy was employed to enhance siRNA delivery in CHO cells, using a TatU1A peptide labeled at the C-terminus with Alexa Fluor 546. Stimulation at 540 nm, which is the excitation wavelength of this Alexa dye, resulted in RNAi-mediated silencing of EGFP in CHO cells and of the epidermal growth factor gene in A431 cells.

### 2.3. Mechanisms of Internalization of S4_13_-PV Peptide

The S4_13_-PV karyophilic cell-penetrating peptide is a synthetic peptide which results from the combination of a 13-amino-acid cell penetrating sequence, derived from the Dermaseptin S4 peptide, with the SV40 (Simian Virus 40) large T antigen nuclear localization signal [23]. Our research group has shown that this peptide accumulates inside live cells and particularly inside the nucleus, through a rapid, dose-dependent and nontoxic process** [57,58].** In addition, and similarly to what has been reported for other CPPs, this peptide has been successfully used to promote intracellular delivery of high molecular weight cargo molecules, in particular DNA, oligonucleotides (ONs) and siRNAs [59].

A comparative study performed recently to assess the internalization capacity of 22 cell-penetrating peptides in four different cell lines has revealed that the S4_13_-PV is one of the peptides exhibiting higher cellular uptake, together with penetratin and transportan [58]. To clarify the mechanisms involved in the internalization of the S4_13_-PV cell-penetrating peptide, we performed a detailed analysis of the cellular uptake and subcellular localization of this peptide in HeLa cells. Results obtained by confocal microscopy and flow cytometry using live cells clearly demonstrated that both low temperatures and depletion of cellular ATP dramatically decreases the number of cells containing the peptide, strongly suggesting that the cellular uptake of S4_13_-PV is mostly mediated by an energy-dependent process [57]. These results are in agreement with other studies performed in live cells, which revealed that some CPPs, such as the Tat peptide, are in fact, internalized mainly by endocytosis [28,29,31]. In this context, the possible involvement of endocytosis in the cellular uptake of the S4_13_-PV peptide was also thoroughly investigated by analyzing peptide uptake in the presence of drugs that selectively compromise different endocytotic pathways, as well as in cells overexpressing a dominant-negative mutant of Dynamin. The fact that S4_13_-PV internalization is not reduced in the presence of these drugs or in the presence of the dynamin-K44A dominant-negative mutant, clearly indicates that endocytosis is not involved in the uptake of this peptide, at least for moderately high S4_13_-PV concentrations (1 µM). In agreement with our results, a recent study confirmed that several inhibitors of the endocytotic pathway, tested in four different cell lines, had little effect on S4_13_-PV internalization [58]. It is important to note that, when we performed studies at very low peptide concentrations (0.1 μM), a reduced uptake of S4_13_-PV was observed upon cell treatment with chloropromazine and nystatin suggesting that, under these experimental conditions, endocytosis may be involved in the internalization of the S4_13_-PV peptide [57].

Similar to what was previously observed with the Tat peptide and Tat fusion proteins [31], a significant inhibition of S4_13_-PV uptake was observed in the presence of low concentrations of heparin [57]. This result suggested that the positively charged peptide has high affinity for the GAG moieties of cell surface proteoglycans. However, comparative analysis of the cellular uptake of S4_13_-PV in normal cells and in cells deficient in proteoglycan biosynthesis showed that although heparan sulphate proteoglycans potentiate the uptake of the S4_13_-PV peptide, their presence at the cell surface is not mandatory for peptide internalization. In fact, the effect of proteoglycans on peptide uptake was shown to be relevant only at low peptide concentrations, whereas at high concentrations almost no differences were observed between cells containing, or not, proteoglycans [57]. 

Altogether, these results demonstrate that two mechanisms are responsible for S4_13_-PV uptake: a GAG- and endocytosis-dependent mechanism, which is dominant at low peptide concentrations, and a GAG- and endocytosis-independent mechanism that occurs preferentially at high peptide concentrations. It is also interesting to note that a recent study performed with the Tat peptide showed that Tat-induced macropinocytosis and peptide uptake occurs efficiently in CHO mutant cells (deficient in heparan sulfate proteoglycans and sialic acid) [46]. These results are in line with our previous results using the S4_13_-PV peptide and together support our interpretation that although acidic proteoglycans favor the binding of certain CPPs, their presence is not a pre-requisite for CPP uptake.

**Figure 2:**
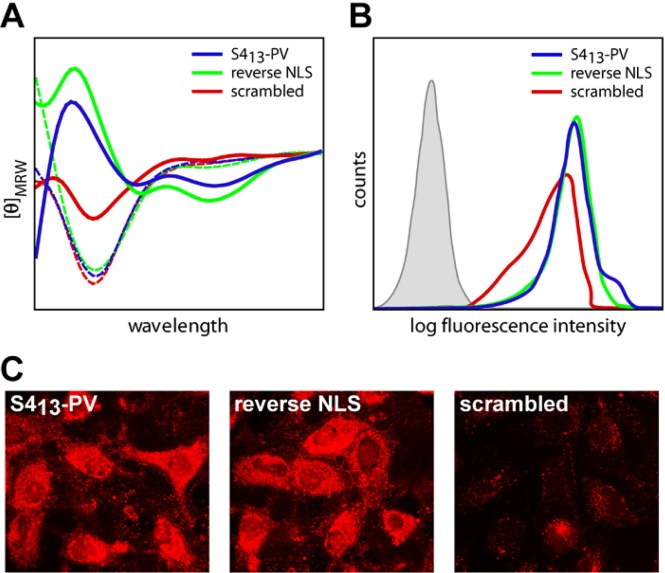
Conformational changes and cellular uptake of the S4_13_-PV, reverse NLS and scrambled peptides. (A) The circular dichroism spectra of the peptides were acquired in sodium phosphate buffer, pH 7.0 (dotted lines), or in the presence of negatively charged membranes composed of POPG, at a lipid/peptide ratio of 4 (straight lines). Clear differences in the peptides spectra was observed in the presence of negatively charged vesicles. (B, C) Hela cells were incubated for 30 minutes, at 37 ºC, with 1.0 μM of rhodamine-labelled peptides. (B) Following treatment with trypsin to remove the non-internalized, surface-bound peptides, cells were analyzed by flow cytometry. (C) Live cells were observed by confocal microscopy. Although all peptides have similar physic-chemical properties, the extent of cellular uptake of the S4_13_-PV and S4_13_-PV reverse NLS peptides was significantly more efficient than that observed for the scrambled peptide.

Aiming at understanding the sequence of events that are the basis of the endocytosis-independent mechanism observed for the S4_13_-PV peptide, which most likely involved direct penetration of the peptide across cell membranes, we performed a detailed biophysical characterization of its interaction with model membranes [60,61]. These studies revealed a clear change in the intrinsic fluorescence spectra of the peptide upon interaction with negatively charged membranes (blue-shift of tryptophan fluorescence), indicating changes of the overall hydrophobicity of the peptide environment. Interestingly, the extent of the blue-shift observed with this peptide was remarkably high when compared with those reported for other CPPs, such as penetratin, transportan [62,63,64] and Pep-1 [65]. Parallel circular dichroism experiments demonstrated that the interaction of the S4_13_-PV peptide with negatively charged membranes induces significant changes in the secondary structure of the peptide (Figure 2), similarly to what has been previously reported for other CPPs, such as penetratin, transportan and Pep-1 [63,65].

In the case of penetratin, it has been shown that low membrane surface charge density favors a mainly helical conformation, while high charge density promotes a dominating β-structure [63,64,66]. In the presence of neutral POPC vesicles, no structure induction takes place relative to the state in aqueous solution [66]. More recently, penetratin has been shown to adopt a helical structure only in the presence of anionic lipids, with the higher structure content observed in the presence of cardiolipin [67]. Pep-1 conformation has been suggested to be helical in the presence of either neutral or charged phospholipids [65], while the MPG peptide was found to be non-ordered in water but to fold into a β-sheet structure upon interacting with phospholipids [68]. In the case of transportan, it has been shown that this peptide adopts a helical structure irrespective of the presence or of the nature of the lipids [63]. In contrast, Tat derived peptide has not been shown to adopt any secondary structure [69].

In our studies, data from circular dichroism analysis showed a general trend towards an increase in the alpha-helical structural motif of the peptide, with increasing membrane charge ratio and lipid/peptide ratio. In addition, studies performed with S4_13_-PV, reverse NLS (S4_13_-PV peptide in which the SV40 NLS sequence is reverted) and scrambled peptides showed that peptide/membrane interactions and peptide uptake seem to be highly dependent on the amino acid sequence of the CPP. Although the initial binding of all peptides to the cell membrane was found to be similar, significant differences were observed in the conformational changes of the S4_13_-PV and reverse NLS peptides as compared to the scrambled peptide, induced upon interaction with the negatively charged target membranes (Figure 2A) [60]. Moreover, a comparative analysis of the cellular uptake of the three peptides, performed by flow cytometry and confocal microscopy (Figure 2B and C), revealed that while the translocation of S4_13_-PV and S4_13_-PV reverse peptides was similar, the uptake of the scrambled peptide was significantly lower [60,61]. 

These results highlight the relevance of the sequence of the S4_13_-PV peptide to the establishment of specific peptide/membrane interactions that occur following its binding to cell membranes, and demonstrated a clear link between the peptide conformational changes and the actual translocation process leading to cellular uptake.

## 3. CPPs-Based Strategies for Delivery of Therapeutic Molecules

Gene-targeted therapies constitute promising approaches for the treatment of numerous pathological conditions, such as cancer, genetic, cardiovascular, inflammatory and infectious diseases, which are characterized by overexpression or inappropriate expression of specific genes [70]. Recent advances in the elucidation of molecular pathways involved in several of these conditions, together with the sequencing of the human genome and the crucial need for innovative and highly specific drugs, with low side effects, have increased the interest on the use of nucleic acids as molecular therapeutics [71]. 

Despite the enormous potential of nucleic acids for the treatment of human diseases, the pharmacological potential of these molecules remains dependent on the development of delivery systems able to mediate their efficient cellular uptake and ensure their correct targeting [7,70,72,73]. Although significant achievements have been made over the years, there is still a clear demand for efficient nucleic acid delivery systems. Ideally, these delivery systems should: (i) protect nucleic acids from degradation; (ii) be effectively internalized in specific target cell types/tissues/organs; (iii) promote release of the carried cargos in the cytoplasm (antisense oligonucleotides, siRNA, miRNA) or nucleus (plasmid DNA, splice-switching oligonucleotides); (iv) exhibit high biological activity at low doses; (v) display no cellular toxicity; and (vi) have a good biosafety profile for *in vivo* therapeutic applications [8]. 

Although the mechanisms underlying the cellular uptake of CPPs and of their conjugates remain highly debated, these peptides have been successfully used to mediate the intracellular delivery of a wide variety of molecules of pharmacological interest in different cell types [11,19,74]. Notably, the relative lack of toxicity and cell specificity have enabled the use of CPP technology in various preclinical models [17].

The ability shared by a considerable number of CPPs to accumulate inside the cell nucleus, render them particularly suited to act as gene delivery vectors. Some CPPs, such as Tat, transportan, polyarginine peptides and S4_13_-PV, have been associated with other non-viral vectors, improving nucleic acid delivery and offering the possibility of combining efficient packaging, delivery and targeting in a single nanocarrier [59,73,75,76,77,78,79,80,81,82,83,84]. The following sections cover the main applications of CPPs in the field of drug delivery, with particular emphasis on the application of CPPs for the delivery of nucleic acids.

### 3.1. Protein Delivery

The use of proteins as therapeutic agents constitutes a very promising approach for the treatment of various diseases. However, the intracellular delivery of these large molecules remains a challenge in part because of their three-dimensional structure, spatial occupation and hydrophilic/hydrophobic nature [85]. Moreover, protein stability relies on weak non-covalent interactions between secondary, tertiary and quaternary structures, which have therefore to be preserved throughout the delivery process [85]. As a result, these molecules appear as highly vulnerable therapeutic agents with short *in vivo* half-lives and poor bioavailability, requiring methods that enable their efficient delivery into cells to be successfully applied *in vivo* [6,85,86]. 

Different types of lipid- and polymer-based vectors have been used for protein delivery, including liposomes, microparticles and nanoparticles, most of them with relatively poor efficiency [85,87]. Alternatively, CPPs have been shown to mediate the delivery of a number of proteins, such as β-galactosidase [88,89], eGFP [90], Bcl-xL [91,92], human catalase [93], human glutamate dehydrogenase [94], Cu,Zn-superoxide dismutase [95], NF-κB inhibitor srIκBα [96] and HSP70 [97], among others. With the exception of Pep-1, a CPP that forms non-covalent complexes with proteins, CPPs are usually coupled to proteins through covalent bonds or through fusion constructs [17,98,99].

Taken together, these studies provide evidence that CPPs are able to mediate the delivery of proteins into a wide variety of cells, both *in vitro* and *in vivo*. Most importantly, these studies demonstrate that CPPs constitute a powerful tool that could be used to facilitate the delivery of protein-based therapeutics in pathological conditions, such as cancer, inflammatory diseases, oxidative stress-related disorders, diabetes and brain injury.

### 3.2. Liposome and Nanoparticle Delivery

Different types of pharmaceutical nanocarriers have been used to increase the stability of drugs, modulate their pharmacokinetics and biodistribution, improve their efficacy and decrease undesired side-effects [73,74]. Numerous attempts have been made to engineer nanosized drug carrier systems, the majority of which based on the use of liposomes and micelles as nanocarriers [73]. These lipid-based vectors, along with nanoparticles, present the possibility of being functionalized with targeting ligands and imaging moieties [73,100,101,102]. CPPs have been used to functionalize liposomes, micelles and nanoparticles, increasing the cellular uptake of the encapsulated cargoes [73,102,103,104,105,106]. These studies emphasize the potential of CPPs in the field of pharmaceutical technology, further demonstrating their versatility and capacity to mediate the delivery of a wide range of molecules, including high molecular weight drugs and drug carriers.

### 3.3. Antisense Oligonucleotide Delivery

The capacity of the antisense technology to target any desired gene and thus modulate a variety of cellular functions is of paramount pharmacological interest [107,108]. This technology is based on the use of sequence specific oligonucleotides (ONs) that, once inside the cells, can hybridize with complementary mRNA strands, causing translational arrest or mRNA degradation through activation of the cellular enzymes of the RNaseH family and consequently blocking gene expression [107]. Among the different ONs with therapeutic potential are: aptamers, transcription factor-binding decoy ONs, ribozymes, triplex-forming ONs, immunostimulatory CpG motifs, antisense ONs, and antagomirs. These ONs can disrupt protein production through three main mechanisms: (i) the formation of an ON/RNA duplex which is a substrate for endogenous RNaseH, leading to mRNA cleavage; (ii) the formation of an ON/mRNA duplex that sterically hinders the assembly of the ribosomal complex or arrests a ribosomal complex already engaged in translation, in both cases affecting protein biosynthesis; (iii) the formation of an ON/mRNA duplex that alters pre-mRNA splicing in the nucleus through a steric-blocking mechanism [72,109]. 

The main advantages of using ONs over protein- or peptide-based approaches are related to their higher target specificity and lower immunogenicity [108]. However, the development of nucleic acid-based therapeutic strategies has been hampered by their poor bioavailability, and therefore the full potential of oligonucleotides as therapeutic agents will not be successfully accomplished if efficient methodologies for targeted delivery to cells and tissues are not developed [110]. Increased stability, enhanced RNA binding affinity and low toxicity are some of the most important aspects to take into account when designing an ON-based approach [107]. Intracellular delivery is also a crucial issue, because in order to affect gene expression by RNaseH-mediated degradation of complementary mRNA, by splicing correction, or by translation arrest, antisense oligonucleotides need to enter the cytoplasm or even the nucleus of cells [110]. These issues have been addressed by chemical modification of oligonucleotides, by using different types of nanocarriers, or by some combination of both strategies [107,108,110,111].

Chemical modification of ONs can drastically improve their stability in the biological environment, their selectivity and biocompatibility [111,112]. Since phosphodiester oligonucleotides are quite unstable, a substitution of sulfur for oxygen, forming phosphorothioate (PS) oligonucleotides, has been commonly performed in order to stabilize both antisense and siRNA molecules [111]. However, as PS oligonucleotides tend to bind nonspecifically to proteins and thereby eliciting toxic effects, other highly improved oligonucleotide chemistries have been developed. These include modifications on the 2’ position of the ribose (2’-O-methyl, 2’-O-methoxy-ethyl, 2’-O-allyl and 2’-O-alkyl), locked nucleic acids (LNAs), peptide nucleic acids (PNAs), phosphorodiamidate morpholino oligomers (PMOs), and hexitol nucleic acids (HNAs) [111,112,113]. Oligonucleotides that include these modifications have improved affinity for mRNA and are more resistant to nuclease degradation, but fail to activate RNaseH-mediated degradation. 

While chemical modifications overcome issues of stability and efficacy, modulation of the pharmacokinetic and biodistribution and, most importantly, improvement of intracellular delivery of oligonucleotides, remain a challenge. Although liposomes and cationic polymers have been successfully applied as a standard tool to deliver ONs into cells *in vitro*, these delivery systems are sometimes characterized by a poor efficiency and associated toxicity when used *in vivo* [108]. 

CPPs have been used for the delivery of ONs by using either a covalent linkage or a non-covalent association to the cargo [9,107,114,115,116]. Steric block small neutral oligonucleotides, including PNAs and PMOs, are potent molecules that have been used for either antisense application or mRNA splicing correction strategies [9]. Several CPPs have been used to mediate the delivery of PNAs and PMOs through covalent linkage of both entities [111,113,114,116,117,118,119]. The formation of efficient non-covalent complexes comprising CPPs and both charged and uncharged steric block oligonucleotides, namely 2'-O-methyl, LNA, PNA and charged PNA derivatives, has also been described [120,121,122,123,124].

Although the first attempt to use ONs to promote inhibition of protein translation was based on the recognition of the DNA:mRNA heteroduplexes by RNaseH leading to RNA cleavage, this approach achieved little clinical success [113]. As a consequence, ONs that are not substrates for RNaseH when hybridized with mRNA have been exploited for the development of alternative therapeutic strategies. An advantage of the use of steric block ONs is their greater specificity, since binding of an ON to an inappropriate mRNA sequence is unlikely to have biological consequences, and thus lower off-target effects are expected when comparing to conventional antisense strategies [113]. Another advantage is the possibility to use a much wider range of synthetic ON analogues than when using conventional antisense approaches, since molecular recognition by RNaseH is not required [113]. Among the great variety of antisense ONs that have been generated, PNA and PMO have come to dominate steric block applications. Despite being neutrally charged, these molecules are as difficult to be internalized by cells as negatively charged ONs [113]. A promising approach towards intracellular delivery of PNAs and PMOs has been their conjugation to CPPs. 

Concerning PNA–CPP conjugates, the first demonstration of the efficacy of this approach consisted in the blocking of expression of the galanin receptor mRNA in human Bowes cells by a 21-mer PNA coupled to penetratin or transportan [117]. In a different study, a model amphipathic peptide (MAP) conjugated to a PNA complementary to the nociceptin/orphanin FQ receptor mRNA was shown to mediate improved cellular uptake and steric block effect in both CHO cells and neonatal rat cardiomyocytes [125]. In order to easily assess the efficiency of nuclear delivery of steric block ONs, a splicing redirection assay described by Kole and coworkers is usually employed [126]. This assay is easy to implement, sensitive, sequence-specific and, most importantly, provides a positive readout over a low background with a large dynamic range. Although a small number of studies reported biological activity when using PNA and PMO coupled to CPPs [117,125,127,128], a considerable number of publications reported that these molecules were only significantly active when in the presence of endosomolytic agents such as chloroquine and calcium ions [129,130,131]. However, most of the existing endosomolytic agents are too toxic to be considered for *in vivo* applications, prompting for the development of CPP-based strategies that are efficient in the absence of these adjuvants. Strategies such as co-treatment with endosome-disrupting peptides [29,132,133] and photochemical internalization [56,134,135,136,137] have been explored. Additionally, a lot of effort has been put on the chemical modification of CPPs, such as poly-arginine, penetratin and transportan 10 (TP10), aiming at rendering these peptides also able to overcome the endosomal mambrane [118,119,121,122]. 

Based on the observation that not all guanidinium side chains of arginine-rich peptides are required for heparin-sulfate binding, arginine residues in poly-arginine peptides have been spaced with non-natural linkers of various lengths and hydrophobicities, aiming at improving the capacity of these peptides to escape from endosomes [119]. For example, both (R-Ahx-R)_4_-PMO [118] and -PNA [138], two modified poly-arginine peptides, were proven to efficiently mediate splice correction in the absence of endosomolytic agents, even if a considerable amount of the conjugates was still trapped in the endocytotic vesicles [71,118,138]. Similar results were obtained when penetratin was modified with arginine residues on its N-terminal. Conjugates of this modified peptide with PNA ONs (R6Pen-PNA conjugates) were more efficient than penetratin itself on promoting splicing redirection [138]. 

Promising results on the *in vivo* use of ONs conjugated with CPPs were obtained in an animal model of Duchenne muscular dystrophy [139,140,141]. The first demonstration of oligonucleotide-mediated exon skipping *in vivo*, was provided by Jearawiriyapaisarn *et al.* [139] using an animal model of Duchenne muscular dystrophy. The potency, functional biodistribution, and toxicity of CPPs containing arginine, 6-aminohexanoic acid, and/or β-alanine conjugated to PMOs were evaluated *in vivo*, in EGFP-654 transgenic mice that ubiquitously express the aberrantly spliced EGFP-654 pre-mRNA [139]. This [139] and other studies, namely by Yin *et al.* [140,141], have shown that a CPP-PMO conjugate restored high-level and uniform dystrophin protein expression in multiple peripheral muscle groups, yielding functional splice correction and improvement of the mdx dystrophic phenotype. 

Although conjugation offers some advantages for *in vivo* applications, such as rationalization, reproducibility of the procedure and control of the stoichiometry of the CPP-cargo conjugates [8], this strategy has also some drawbacks such as the possibility to compromise the biological activity of the cargo [8] and the need to generate and test a new construct for any given nucleic acid cargo [108]. Non-covalent strategies appear to be, therefore, more promising, especially in the case of negatively-charged ONs, which can readily interact with positively charged CPPs. As an example, Morris *et al.* [123], described in 2004 a novel technology that non-covalently combined a new generation of PNAs (HypNApPNAs) with Pep-2, which resulted in efficient delivery of PNAs into several cell lines. A similar strategy was described for Pep-3, which was found to form stable complexes with both uncharged and charged PNAs, and to promote their cellular uptake in different cell lines [124]. Of notice, it was demonstrated that Pep-3-mediated delivery of antisense-cyclin B1-charged-PNA inhibits tumor growth *in vivo* upon intratumoral or intravenous injection [124]. In addition, it was shown that PEGylation of Pep-3 significantly improved complex stability *in vivo* and consequently the efficiency of cyclin B1 anisense ONs, when administered intravenously.

More recently, stearylated analogs of (RxR)_4_ and TP10 were shown to interact with negatively charged ONs and to promote their efficient delivery into cells [121,122]. Importantly, the stearic acid modification of these peptides was associated with increased endosomal escape. MPGα has also been used to mediate the uptake of different chemically modified (2'-O-methyl, LNA and PNA) steric block oligonucleotides [120]. 

Our attempts to use the S4_13_-PV peptide for the delivery of splice correcting ONs have shown that S4_13_-PV/ONs complexes were internalized by cells but localized primarily inside the endosomes [142]. These complexes presented no biological activity even in the presence of different endosomolytic agents, indicating that endosomal entrapment is not the only factor hampering the efficient delivery of the ONs to their target sites. Interestingly, the combination of the S4_13_-PV and reverse NLS peptides with a previously developed lipoplex-based formulation (DLS) resulted in an efficient, specific and non-cytotoxic system for mediating splice correction, superior to that obtained either by the DLS-based system or the covalent conjugate R(Ahx)R_4_-PMO [142]. 

Altogether, these results emphasize the need for improved solutions regarding endosomal escape, while strongly suggesting that the use of CPPs constitute a very promising approach for the delivery of ONs. 

### 3.4. siRNA Delivery

RNA interference (RNAi) has become an indispensable tool for studying gene functions and constitutes an attractive approach for the development of novel therapeutic strategies for pathological disorders [143,144,145,146,147]. However, siRNAs share the same delivery problems as DNA ONs, which has so far limited their therapeutic application [146,147]. Although a considerable number of viral and non-viral strategies have been designed to overcome such limitations, clinically viable siRNA delivery approaches have not been developed to date [147,148]. 

CPPs have been used for delivery of siRNAs either by covalent or non-covalent approaches, similarly to what was previously described for ONs [7,9,149]. The preparation of non-covalent complexes between siRNAs and the CPPs is technically simpler, originating aggregates or nanoparticles with a net positive charge [7]. On the other hand, the covalent linkage of CPPs to siRNAs allows the formation of small, monomeric CPP/siRNA conjugates of known stoichiometry with high reproducibility [7,8].

Efficient delivery of siRNAs has been reported by their covalent association with transportan [150], Tat [151] and penetratin [152]. However, in these studies, the CPP/siRNA conjugate was added to cells without a purification step following the cross-linking procedure [7,149], raising a doubt as to whether the successful delivery described in these conditions results from the CPP/siRNA covalent conjugates, or rather from non-covalent complexes formed between excess free peptide and siRNAs [7,127,148,153,154]. Meade *et al.* [153] observed that after extensive purification of CPP/siRNA conjugates from the excess cationic peptide in the conjugation reaction, no enhanced cellular internalization of siRNA could be detected. Additionally, it has been shown that silencing of an endogenous gene by certain CPP/siRNA conjugates requires very high levels of the conjugate - a thousand-fold or more than that typically used in lipofection [127]. Of note, the concentration of CPP/siRNA conjugates required in this study to achieve significant protein knockdown was considerably higher than those used in previously published studies, which employed non-purified CPP/siRNA [150,151,152]. For these reasons, non-covalent strategies are usually preferred for siRNA delivery [7,84,155,156,157,158,159]. 

One of the first reports of non-covalent approach for the delivery of siRNAs involved their stable complexation with the MPG peptide, a peptide derived from the combination of the hydrophobic fusion peptide of HIV-1 gp41 and the hydrophilic nuclear localization sequence of SV40 large T antigen [155]. Although a significant downregulation of the target protein was achieved using this peptide (ca. 80% reduction in protein activity), a mutation in the NLS sequence of the carrier peptide (MPGΔNLS), that was intended to favor rapid release of the siRNA into the cytoplasm, further increased the RNAi effect [155]. This peptide was applied *in vivo* for delivery of siRNAs targeting OCT-4 into mouse blastocytes [160], as well as for silencing cyclin B1 [161]. In the latter study, MPG/siRNA complexes were shown to prevent tumor growth in mice after systemic administration [161]. A variant of MPG (MPGα), which comprises five mutations in its hydrophobic domain that favor an alpha-helical conformation of the peptide, has also been shown to efficiently mediate siRNA delivery [55].

Polyarginine peptides have also been exploited for the delivery of siRNA. In 2006, Kim and coworkers synthesized a cholesteryl oligo-arginine (nine residues) conjugate – Chol-R9 – as a siRNA delivery vehicle, which was used to mediate the silencing of vascular endothelial growth factor (VEGF) [159]. More recently, a chimaeric peptide was synthesized by adding nine arginine residues at the carboxy terminus of the RVG peptide [84]. *In vitro* studies demonstrated that the RVG-9R peptide was able to bind to siRNAs and transduce neuronal cells resulting in efficient gene silencing [84]. Moreover, intravenous administration of these complexes into mice led to a specific gene silencing in the central nervous system [84]. 

A non-covalent strategy using an endosomolytic CPP based on penetratin was described by Lundberg *et al.* [156]. In this study, the peptide EB1 was found to be far more effective both in forming complexes and transporting biologically active siRNA than its parental peptide penetratin [156]. It is important to mention that, in this study, other CPPs besides penetratin and EB1 were evaluated in terms of complex formation, cellular uptake and gene silencing (MPG-ΔNLS, TP-10, Bovine PrP 1-30 and HA2-penetratin). It was demonstrated that even though all of the CPPs evaluated could form complexes with siRNAs, no direct association between the complex formation ability and delivery efficacy could be established [156]. Moreover, although all CPPs significantly promoted cellular uptake of the siRNAs, gene silencing effect was critically dependent on endosomal escape of the internalized complexes [156].

An alternative non-covalent approach, which is not based on the formation of electrostatic interaction between the cationic CPPs and the negatively charged siRNAs, has been recently proposed by Eguchi *et al.* [158]. The strategy involves the generation of a chimearic peptide composed of a dsRNA binding domain (DRBD) fused to a Tat-based PTD. The DRBD portion of the peptide is responsible for binding to siRNA with high affinity, masking its negative charges, while the Tat moiety promotes the intracellular delivery of the PTD–DRBD siRNA complex [158]. The PTD-DRBD-siRNA complexes induced a rapid and efficient silencing of the target gene in a large percentage of primary and transformed cells, including T cells, human umbilical vein endothelial cells and human embryonic stem cells, with no apparent cytotoxicity, minimal off-target transcriptional changes and no induction of innate immune responses [158]. 

Our own observations using the S4_13_-PV peptide are in line with the results described by Lundberg *et al.* for other CPPs [156]. Although the S4_13_-PV peptide is able to form non-covalent complexes through electrostatic association with siRNAs, the resulting complexes do not mediate significant protein knockdown in a cell line stably expressing GFP (Trabulo, S, Cardoso, AM, Cardoso, AL, and Pedroso de Lima, MC, unpublished observations). However, under the same experimental conditions, complexes prepared with fluorescently labeled siRNAs were efficiently internalized, strongly suggesting that endosomal entrapment is the major hurdle limiting siRNA-mediated gene silencing by these complexes (Trabulo, S, Cardoso, AM, Cardoso, AL, and Pedroso de Lima, MC, unpublished observations). Further supporting this hypothesis, we observed that combination of the S4_13_-PV/siRNA complexes with cationic liposomes containing DOPE, a fusogenic lipid that has been shown to facilitate endosomal release of lipoplexes [162,163], mediated GFP knockdown as efficiently as complexes prepared with Lipofectamine-2000 (Trabulo, S, Cardoso, AM, Cardoso, AL, and Pedroso de Lima, MC, unpublished observations).

A study by Zhang *et al.* [83] also described a combination of CPPs and liposomes in which an arginine octamer (R8) was attached to the surface of the liposomes encapsulating siRNAs. This system demonstrated to be stable and able to efficiently mediate gene silencing in all the tested lung tumor cell lines, while presenting low non-specific toxicity [83].

Despite many hurdles, development of siRNA-based therapeutics has advanced rapidly over the last few years. However, the major challenge limiting the widespread application of this technology is still delivery efficiency [147]. Although at least five clinical trials are already ongoing, these trials involve the administration of saline-formulated siRNA rather than the described conjugates or non-covalent complexes [164]. This indicates that despite the great progresses that have been made in the field of nucleic acid delivery, much remains to be done. Issues concerning safety, scale-up, reproducibility, analytical characterization and pharmaceutical acceptability should not be overlooked when a clinical application is sought [111,164].

Nevertheless, the studies described show encouraging results by using easy and versatile strategies to deliver siRNAs. CPPs are certainly among the most promising candidates to be used in the development of siRNA-based therapeutics. 

### 3.5. Gene Delivery

The main goal of gene therapy consists in delivering therapeutic genes into the nucleus of target cells to achieve expression of a deficient or incorrectly expressed gene product [165]. As for the other types of biomolecules described so far, difficulties in developing safe and efficient gene delivery vectors able to sustain gene expression for long periods has limited a broader clinical application of gene delivery [165]. 

Viral vectors present certain advantages in the context of gene delivery, including high and sustained levels of transduction and in some cases efficient and stable integration of exogenous DNA into a wide range of host genomes [166,167]. However, this type of vectors also present several problems, such as immunogenicity, toxicity, difficulty of large-scale production, size limit of the exogenous DNA, insertional mutagenesis caused by random integration into the host genome, and the risks of inducing oncogenic mutations or generating active viral particles through recombination mechanisms [165,166,168]. These limitations of viral vectors justify the interest on the development of improved non-viral gene delivery vectors.

The most commonly used non-viral vectors for plasmid DNA delivery are cationic liposomes, nanoparticles, cationic polymers, and CPPs [167,168,169]. The use of cationic peptides for gene delivery is particularly interesting because they are able to efficiently condense DNA due to electrostatic interaction, can be attached to liposomes or polymers allowing for efficient targeting, are able to improve cellular internalization and promote endosomal escape and can provide nuclear localization of condensates when short NLS peptides are used [8,73,168]. 

There are many examples of CPP-mediated delivery of plasmid DNA into cultured cells and also *in vivo* involving the use of a non-covalent approach [72]. While some approaches involve single-component peptide vectors, the major focus has been on the association of CPPs with other non-viral gene delivery methods, such as liposomes, polyethyleneimine (PEI) or nanoparticles.

In 1999, Morris *et al.* [170] demonstrated that MPG could be used as a powerful tool for the delivery of nucleic acids. It was shown that MPG is not cytotoxic, insensitive to serum and able to efficiently deliver plasmid DNA into several different cell lines [170]. Further studies demonstrated that cell entry of the MPG/DNA particles is independent of the endosomal pathway and that the NLS of MPG is involved in both electrostatic interactions with DNA and nuclear targeting [155]. Furthermore, it was shown that a mutation affecting the NLS of MPG prevents nuclear delivery of DNA [155].

In an alternative study, Rittner *et al.* [171] described the novel basic amphiphilic peptides, ppTG1 and ppTG20 (20 amino acids), and evaluated their efficiencies *in vitro* and *in vivo* as single-component gene transfer vectors. It was demonstrated that both the ppTG1 and ppTG20 peptides are able to bind nucleic acids and destabilize membranes, in a liposome leakage assay [171]. Complexes of plasmid DNA with ppTG1 originated high levels of gene expression in cell culture experiments and, most importantly, complexes of plasmid DNA with ppTG1 or ppTG20 led to significant gene expression *in vivo* [171].

Peptide modification has also been explored as a means to enhance gene delivery. In particular, stearic acid modification of different membrane-permeable arginine-rich peptides, such as HIV-1 Tat (48-60), HIV-1 Rev (34-50), flock house virus (FHV) coat (35-49), (RxR)_4_ and oligoarginines of 4-16 residues was shown to substantially increase their transfection efficiency [121,172,173]. The mechanisms by which stearic acid modification improves plasmid DNA delivery by CPPS have been shown to involve increased efficiency of endosomal escape [121] or enhanced cellular association, as well as higher nuclear delivery [173].

The extensively studied Tat peptide has also been exploited for plasmid DNA delivery by different research groups, with contradictory results. A study by Ignatovich *et al.* [77], demonstrated that Tat peptide is able to form complexes with plasmid DNA, which could be used for gene delivery into mammalian cells. Despite reasonably high transfection efficiency *in vitro*, low gene expression levels were detected in the liver of mice injected intravenously with DNA-Tat complexes, a fact that was attributed to inactivation of the complexes in the bloodstream due to interactions with serum albumin [77]. Interestingly, an endocytosis-dependent mechanism was proposed for the uptake of the DNA-Tat complexes, similar to what was proposed for internalization of complexes of plasmid DNA with other polycationic carriers [77]. A different study, by Tung *et al.* [81], compared the efficiency of a series of ramified Tat peptides, containing 1–8 Tat moieties. Although all compounds complexed with plasmid DNA, it was demonstrated that at least eight Tat peptide moieties are required in order to achieve efficient gene delivery [81]. Sandgren *et al.* [174] also studied the cellular uptake of complexes of plasmid DNA and the HIV-Tat derived peptide. According to this study, the Tat peptide stimulated cellular uptake of DNA in a time-, concentration-, and temperature-dependent manner, while accumulating in large, acidic, cytoplasmic vesicles, followed by transfer of the cargo into the nuclear compartment and subsequent disappearance from the endolysosomal vesicles [174]. Aiming at increasing the efficiency of the Tat peptide to deliver plasmid DNA, Lo *et al.* [175] made several modifications to the Tat peptide, through the use of histidine and cysteine residues to enhance endosomal escape and complex stability. Up to 7,000-fold improvement in gene transfection efficiency was observed for the Tat peptide covalently fused with 10 histidine residues (Tat-10H) over the original Tat peptide, and incorporation of two cysteine residues into this peptide resulted in an even higher efficacy (C-5H-Tat-5H-C) [175].

The association of CPPs with other non-viral delivery vectors has also been extensively investigated, aiming at exploring the possibility to combine efficient delivery, packaging and targeting moieties within the same system [176,177].

A combination of a PNA with the SV40 core NLS, performed by Branden *et al.* [178], originated a bifunctional peptide that improved the efficacy of plasmid transfection up to 8-fold when associated with the transfection agent polyethyleneimine (PEI). Several other studies also combined PEI with CPPs [75,76,179]. Kleemann *et al.* [179] covalently coupled the Tat peptide to 25 kDa PEI through a heterobifunctional polyethylenglycol (PEG) spacer resulting in a Tat-PEG-PEI conjugate. Improved DNA reporter gene complexation and protection were observed for small (approximately 90 nm) polyplexes as well as low toxicity and significantly enhanced transfection efficiency *in vivo* [179].

Rudolph *et al.* [76] demonstrated that oligomers of the Tat peptide were able to condense plasmid DNA to nanosized particles and protect DNA from nuclease degradation. Most importantly, when DNA was pre-condensed with Tat peptides and PEI, Superfect or LipofectAMINE were added to the mixture, transfection efficiency was enhanced up to 390-fold compared with the standard vectors [76]. Similar studies by Kilk *et al.* [75], demonstrated that the poor transfection abilities exhibited by TP10 was significantly enhanced in the presence of PEI, increasing several fold compared to PEI alone, particularly at low PEI concentrations, therefore allowing the use of reduced PEI concentration [75].

The association of lipid-based vectors with CPPs has been the subject of a number of studies, including those from our group [59,78,80,82,105,180,181,182,183]. Using fluorescently labeled liposomes and cargos, Torchilin *et al.* demonstrated that large drug carriers, such as 200-nm liposomes, could be delivered into cells by attaching Tat peptide to the liposome surface [180]. Later, the same group described the formation of non-covalent complexes of Tat, liposomes and DNA that were able to efficiently transfect cells both *in vitro* and *in vivo,* while being less toxic than other commonly used transfection reagents [105]. The internalization of this system was claimed to rely on a direct cytoplasmic delivery imparted by the Tat peptide [105].

A study by Hyndman *et al.* [182] showed that mixing the CPP Tat with liposomes containing DOTAP or Lipofectin and DNA, resulted in complexes that significantly enhance transfection *in vitro* with a marked reduction in the amount of liposomes required, despite the lack of any covalent linkage of the peptide to liposomes. In this study, the use of endosomolytic agents and results from experiments performed at low temperature suggested that the endocytotic pathway was involved in the internalization of the complexes [182]. Another report demonstrated that the increase in gene transfer of Tat-modified lipoplexes is dependent on the amount of cationic lipid in the lipoplexes and on the way Tat was coupled to the lipoplexes [82]. Moreover, it was shown that the cellular uptake of both Tat-modified and unmodified lipoplexes was very fast and, in contrast to previous publications, temperature-dependent [82].

A concept called “Programmed Packaging” was proposed by Kogure *et al.* [78], who developed a Multifunctional Envelope-type Nano Device (MEND), consisting of a condensed DNA core and a surrounding lipid envelope. This packaging method involves three steps: (i) DNA condensation with a polycation, (ii) lipid film hydration for the electrostatic binding of the condensed DNA, and (iii) sonication to package the condensed DNA with lipids [78]. MEND, having octa-arginine on the envelope as a mean to enhance cellular uptake, showed a 1,000-fold higher transfection activity than a DNA/poly-L-lysine/lipid complex prepared in similar conditions [78]. Another study, by Khalil *et al.* [80], also described the high-efficiency delivery of nucleic acids to eukaryotic cells using MEND particles containing polycation-condensed nucleic acids encapsulated in an R8-DOPE lipid envelope. MEND particles were shown to be non-cytotoxic and achieved transfection efficiencies as high as adenovirus [80]. In this case, the high efficiency of MEND particles was attributed, at least in part, to R8 which was claimed to promote cellular uptake by macropinocytosis, improving intracellular trafficking towards more efficient gene expression [80]. Along the same lines, work of the same research group [184] demonstrated that gene expression of condensed plasmid DNA encapsulated in R8-modified nanoparticles was more than one order of magnitude higher than that of K8-modified nanoparticles, and two orders of magnitude higher than gene expression using unmodified nanoparticles. Differences in gene expression achieved with R8- and K8-modified liposomes could not be attributed to differences in cellular uptake, since both kinds of complexes were taken up primarily *via* macropinocytosis at comparable efficiencies [184]. Moreover, it was described that modification of nanoparticles with a high density of R8 allows their escape from endocytotic vesicles *via* membrane fusion at both acidic and neutral pH, and that the guanidinium groups of arginine residues, and not only their positive charge, are important for efficient endosomal escape [184].

Recently, MacKay *et al.* [181] described gene transfer using PEGylated bioresponsive nanolipid particles (NLPs) containing plasmid DNA. In this study, the Tat peptide was attached either directly to a phospholipid (Tatp-lipid) or *via* a 2-kDa polyethylene glycol (PEG) (Tatp-PEG-lipid); incorporation of 0.3 mol% Tatp-PEG into pH-sensitive NLPs improved transfection 100,000-fold compared to NLPs [181]. Although Tatp-PEG-lipid could dramatically increase gene expression *in vitro*, when tested in brain and in implanted tumors, a restriction of NLP distribution to the vicinity of the infusion catheter reduced the absolute level of gene transfer [181]. 

**Figure 3:**
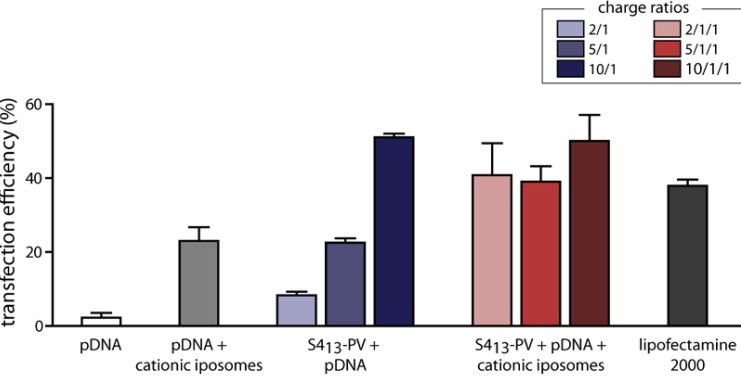
Efficiency of transfection mediated by different complexes containing the S4_13_-PV peptide. HeLa cells were incubated with free plasmid DNA, cationic liposome/DNA complexes, Lipofectamine 2000-based complexes and the ternary complexes for 4 h at 37 ºC. Transfection efficiency was evaluated, 48 h later, by flow cytometry analysis of GFP expression and the percentage of GFP-positive cells is presented. Ternary complexes were obtained by the addition of cationic liposomes composed of DOTAP:DOPE to complexes of S4_13_-PV, prepared at different peptide/DNA charge ratios. Ternary complexes were able to transfect cells more efficiently than cationic liposome/DNA complexes and at similar levels than those obtained with Lipofectamine 2000.

In our studies [59], complexes obtained through electrostatic association of the S4_13_-PV cell-penetrating peptide with plasmid DNA are able to very efficiently mediate transfection, particularly at high peptide/DNA charge ratios (5/1 and higher). Importantly, complexes prepared with the S4_13_-PV or reverse NLS peptides mediate transfection at significantly higher efficiencies than those containing the scrambled version of the peptide, demonstrating the importance of the cell-penetrating sequence derived from the Dermaseptin S4 peptide (amino acids 1–13) to the transfection process [59]. Additionally, we demonstrated that ternary complexes, resulting from association of cationic liposomes to peptide/DNA complexes, are significantly more efficient in mediating transfection than the corresponding peptide/DNA or cationic liposome/DNA complexes (Figure 3) [59]. 

In agreement with what has been described for oligonucleotides, CPPs seem to be very efficient to mediate the uptake of plasmid DNA, as well as lipoplexes and polyplexes containing DNA, surpassing the cell membrane barrier. However, the challenge of overcoming the entrapment of complexes inside endosomes has not been solved as easily as initially anticipated, even taking advantage of the capacity of direct translocation to the cytoplasm of some CPPs. Nevertheless, several of the studies described above present promising strategies to overcome this limitation, such as chemical modification of the peptide backbone or coupling of CPPs to other classes of delivery vectors. Overall, accumulated evidence suggests that CPPs used in combination with other delivery systems are more likely to be effective for gene therapy purposes than CPPs alone.

## 4. Conclusions

Research on CPPs as drug delivery systems has clarified their capacity to promote the efficient internalization of therapeutic biomolecules. Despite differences in size, charge and/or structure between different bioactive molecules, it seems clear that CPP-based systems appear to be very versatile and efficient delivery is achievable following proper adjustment of the carrier to the transported biomolecule.

Because the development of drug, oligonucleotide or gene delivery systems is aimed at a clinical application, the design of these innovative delivery vectors should consider other important issues, beyond *in vitro* and *vivo* demonstration of efficacy, which include safety, biodistribution, ease of manufacturing, scale-up, reproducibility and analytical and physical characterization. Once such issues are properly addressed, these new-generation systems will undoubtedly find their place in successful gene therapies. 

In our opinion, the advance of CPP technology depends on the development of strategies that facilitate endosomal escape and that confer cell specificity to these systems. From what has been discussed in this review, these studies are already ongoing and promising results have already been reported. A careful investigation of the mechanisms of internalization of CPP-cargo complexes or conjugates, along with a better understanding of the complex network of endocytotic pathways will greatly help the improvement of this powerful technology. 

## References

[B1-pharmaceuticals-03-00961] Lindgren M., Hallbrink M., Prochiantz A., Langel U. (2000). Cell-penetrating peptides. Trends Pharmacol. Sci..

[B2-pharmaceuticals-03-00961] Frankel A.D., Pabo C.O. (1988). Cellular uptake of the tat protein from human immunodeficiency virus. Cell.

[B3-pharmaceuticals-03-00961] Green M., Loewenstein P.M. (1988). Autonomous functional domains of chemically synthesized human immunodeficiency virus tat trans-activator protein. Cell.

[B4-pharmaceuticals-03-00961] Joliot A.H., Triller A., Volovitch M., Pernelle C., Prochiantz A. (1991). alpha-2,8-Polysialic acid is the neuronal surface receptor of antennapedia homeobox peptide. New Biol..

[B5-pharmaceuticals-03-00961] Derossi D., Joliot A.H., Chassaing G., Prochiantz A. (1994). The third helix of the Antennapedia homeodomain translocates through biological membranes. J. Biol. Chem..

[B6-pharmaceuticals-03-00961] Morris M.C., Depollier J., Mery J., Heitz F., Divita G. (2001). A peptide carrier for the delivery of biologically active proteins into mammalian cells. Nat. Biotechnol..

[B7-pharmaceuticals-03-00961] Meade B.R., Dowdy S.F. (2007). Exogenous siRNA delivery using peptide transduction domains/cell penetrating peptides. Adv. Drug Deliv. Rev..

[B8-pharmaceuticals-03-00961] Heitz F., Morris M.C., Divita G. (2009). Twenty years of cell-penetrating peptides: from molecular mechanisms to therapeutics. Br. J. Pharmacol..

[B9-pharmaceuticals-03-00961] Morris M.C., Deshayes S., Heitz F., Divita G. (2008). Cell-penetrating peptides: from molecular mechanisms to therapeutics. *Biol*. Cell.

[B10-pharmaceuticals-03-00961] Langel U. (2002). Cell Penetrating Peptides: Processes and Applications.

[B11-pharmaceuticals-03-00961] Dietz G.P., Bahr M. (2004). Delivery of bioactive molecules into the cell: the Trojan horse approach. Mo.l Cell Neurosci..

[B12-pharmaceuticals-03-00961] Magzoub M., Graslund A. (2004). Cell-penetrating peptides: [corrected] from inception to application. Q. Rev. Biophys..

[B13-pharmaceuticals-03-00961] Joliot A., Prochiantz A. (2004). Transduction peptides: from technology to physiology. Nat. Cell Biol..

[B14-pharmaceuticals-03-00961] Deshayes S., Morris M.C., Divita G., Heitz F. (2005). Cell-penetrating peptides: tools for intracellular delivery of therapeutics. Cell. Mol. Life. Sci..

[B15-pharmaceuticals-03-00961] Vives E. (2005). Present and future of cell-penetrating peptide mediated delivery systems: "is the Trojan horse too wild to go only to Troy?". J. Control. Release.

[B16-pharmaceuticals-03-00961] El-Andaloussi S., Holm T., Langel U. (2005). Cell-penetrating peptides: mechanisms and applications. Curr. Pharm. Des..

[B17-pharmaceuticals-03-00961] Patel L.N., Zaro J.L., Shen W.C. (2007). Cell Penetrating Peptides: Intracellular Pathways and Pharmaceutical Perspectives. Pharm. Res..

[B18-pharmaceuticals-03-00961] Wagstaff K.M., Jans D.A. (2006). Protein transduction: cell penetrating peptides and their therapeutic applications. Curr. Med. Chem..

[B19-pharmaceuticals-03-00961] Gupta B., Levchenko T.S., Torchilin V.P. (2005). Intracellular delivery of large molecules and small particles by cell-penetrating proteins and peptides. Adv. Drug. Deliv. Rev..

[B20-pharmaceuticals-03-00961] Lundberg M., Wikstrom S., Johansson M. (2003). Cell surface adherence and endocytosis of protein transduction domains. Mol. Ther..

[B21-pharmaceuticals-03-00961] Richard J.P., Melikov K., Vives E., Ramos C., Verbeure B., Gait M.J., Chernomordik L.V., Lebleu B. (2003). Cell-penetrating peptides. A reevaluation of the mechanism of cellular uptake. J. Biol. Chem..

[B22-pharmaceuticals-03-00961] Vives E., Richard J.P., Rispal C., Lebleu B. (2003). TAT peptide internalization: seeking the mechanism of entry. Curr. Protein Pept. Sci..

[B23-pharmaceuticals-03-00961] Hariton-Gazal E., Feder R., Mor A., Graessmann A., Brack-Werner R., Jans D., Gilon C., Loyter A. (2002). Targeting of nonkaryophilic cell-permeable peptides into the nuclei of intact cells by covalently attached nuclear localization signals. Biochemistry.

[B24-pharmaceuticals-03-00961] Derossi D., Calvet S., Trembleau A., Brunissen A., Chassaing G., Prochiantz A. (1996). Cell internalization of the third helix of the Antennapedia homeodomain is receptor-independent. J. Biol. Chem..

[B25-pharmaceuticals-03-00961] Huq I., Ping Y.H., Tamilarasu N., Rana T.M. (1999). Controlling human immunodeficiency virus type 1 gene expression by unnatural peptides. Biochemistry.

[B26-pharmaceuticals-03-00961] Wender P.A., Mitchell D.J., Pattabiraman K., Pelkey E.T., Steinman L., Rothbard J.B. (2000). The design, synthesis, and evaluation of molecules that enable or enhance cellular uptake: peptoid molecular transporters. Proc. Natl. Acad. Sci. U. S. A..

[B27-pharmaceuticals-03-00961] Ferrari M.E., Nguyen C.M., Zelphati O., Tsai Y., Felgner P.L. (1998). Analytical methods for the characterization of cationic lipid-nucleic acid complexes. Hum. Gene Ther..

[B28-pharmaceuticals-03-00961] Fittipaldi A., Ferrari A., Zoppe M., Arcangeli C., Pellegrini V., Beltram F., Giacca M. (2003). Cell membrane lipid rafts mediate caveolar endocytosis of HIV-1 Tat fusion proteins. J. Biol. Chem..

[B29-pharmaceuticals-03-00961] Wadia J.S., Stan R.V., Dowdy S.F. (2004). Transducible TAT-HA fusogenic peptide enhances escape of TAT-fusion proteins after lipid raft macropinocytosis. Nat. Med..

[B30-pharmaceuticals-03-00961] Kaplan I.M., Wadia J.S., Dowdy S.F. (2005). Cationic TAT peptide transduction domain enters cells by macropinocytosis. J. Control. Release.

[B31-pharmaceuticals-03-00961] Richard J.P., Melikov K., Brooks H., Prevot P., Lebleu B., Chernomordik L.V. (2005). Cellular uptake of unconjugated TAT peptide involves clathrin-dependent endocytosis and heparan sulfate receptors. J. Biol. Chem..

[B32-pharmaceuticals-03-00961] Belting M. (2003). Heparan sulfate proteoglycan as a plasma membrane carrier. Trends Biochem. Sci..

[B33-pharmaceuticals-03-00961] Fuchs S.M., Raines R.T. (2004). Pathway for polyarginine entry into mammalian cells. Biochemistry.

[B34-pharmaceuticals-03-00961] Berlose J.P., Convert O., Derossi D., Brunissen A., Chassaing G. (1996). Conformational and associative behaviours of the third helix of antennapedia homeodomain in membrane-mimetic environments. Eur. J. Biochem..

[B35-pharmaceuticals-03-00961] Pouny Y., Rapaport D., Mor A., Nicolas P., Shai Y. (1992). Interaction of antimicrobial dermaseptin and its fluorescently labeled analogues with phospholipid membranes. Biochemistry.

[B36-pharmaceuticals-03-00961] Shai Y. (1999). Mechanism of the binding, insertion and destabilization of phospholipid bilayer membranes by alpha-helical antimicrobial and cell non-selective membrane-lytic peptides. Biochim. Biophys. Acta.

[B37-pharmaceuticals-03-00961] Matsuzaki K., Sugishita K., Miyajima K. (1999). Interactions of an antimicrobial peptide, magainin 2, with lipopolysaccharide-containing liposomes as a model for outer membranes of gram-negative bacteria. FEBS Lett..

[B38-pharmaceuticals-03-00961] Lundberg P., Langel U. (2003). A brief introduction to cell-penetrating peptides. J. Mol. Recognit..

[B39-pharmaceuticals-03-00961] Yang L., Harroun T.A., Weiss T.M., Ding L., Huang H.W. (2001). Barrel-stave model or toroidal model? A case study on melittin pores. Biophys. J..

[B40-pharmaceuticals-03-00961] Conner S.D., Schmid S.L. (2003). Regulated portals of entry into the cell. Nature.

[B41-pharmaceuticals-03-00961] Mayor S., Pagano R.E. (2007). Pathways of clathrin-independent endocytosis. Nat. Rev. Mol. Cel.l Biol..

[B42-pharmaceuticals-03-00961] Duchardt F., Fotin-Mleczek M., Schwarz H., Fischer R., Brock R. (2007). A comprehensive model for the cellular uptake of cationic cell-penetrating peptides. Traffic.

[B43-pharmaceuticals-03-00961] Fischer R., Fotin-Mleczek M., Hufnagel H., Brock R. (2005). Break on through to the other side-biophysics and cell biology shed light on cell-penetrating peptides. Chembiochem.

[B44-pharmaceuticals-03-00961] Ferrari A., Pellegrini V., Arcangeli C., Fittipaldi A., Giacca M., Beltram F. (2003). Caveolae-mediated internalization of extracellular HIV-1 tat fusion proteins visualized in real time. Mol. Ther..

[B45-pharmaceuticals-03-00961] Nakase I., Niwa M., Takeuchi T., Sonomura K., Kawabata N., Koike Y., Takehashi M., Tanaka S., Ueda K., Simpson J.C., Jones A.T., Sugiura Y., Futaki S. (2004). Cellular uptake of arginine-rich peptides: roles for macropinocytosis and actin rearrangement. Mol. Ther..

[B46-pharmaceuticals-03-00961] Gump J.M., June R.K., Dowdy S.F. (2009). Revised role of glycosaminoglycans in TAT PTD-mediated cellular transduction. J. Biol. Chem..

[B47-pharmaceuticals-03-00961] Ter-Avetisyan G., Tunnemann G., Nowak D., Nitschke M., Herrmann A., Drab M., Cardoso M.C. (2009). Cell entry of arginine-rich peptides is independent of endocytosis. J. Biol. Chem..

[B48-pharmaceuticals-03-00961] Jones A.T. (2008). Gateways and tools for drug delivery: endocytic pathways and the cellular dynamics of cell penetrating peptides. Int. J. Pharm..

[B49-pharmaceuticals-03-00961] Lundin P., Johansson H., Guterstam P., Holm T., Hansen M., Langel U., S E.L.A. (2008). Distinct uptake routes of cell-penetrating peptide conjugates. Bioconjug. Chem..

[B50-pharmaceuticals-03-00961] Soomets U., Lindgren M., Gallet X., Hallbrink M., Elmquist A., Balaspiri L., Zorko M., Pooga M., Brasseur R., Langel U. (2000). Deletion analogues of transportan. Biochim. Biophys. Acta.

[B51-pharmaceuticals-03-00961] Caron N.J., Quenneville S.P., Tremblay J.P. (2004). Endosome disruption enhances the functional nuclear delivery of Tat-fusion proteins. Biochem. Biophys. Res. Commun..

[B52-pharmaceuticals-03-00961] Seglen P.O., Grinde B., Solheim A.E. (1979). Inhibition of the lysosomal pathway of protein degradation in isolated rat hepatocytes by ammonia, methylamine, chloroquine and leupeptin. Eur. J. Biochem..

[B53-pharmaceuticals-03-00961] Maiolo 3rd J.R., Ottinger E.A., Ferrer M. (2004). Specific redistribution of cell-penetrating peptides from endosomes to the cytoplasm and nucleus upon laser illumination. J. Am. Chem. Soc..

[B54-pharmaceuticals-03-00961] Matsushita M., Noguchi H., Lu Y.F., Tomizawa K., Michiue H., Li S.T., Hirose K., Bonner-Weir S., Matsui H. (2004). Photo-acceleration of protein release from endosome in the protein transduction system. FEBS Lett..

[B55-pharmaceuticals-03-00961] Veldhoen S., Laufer S.D., Trampe A., Restle T. (2006). Cellular delivery of small interfering RNA by a non-covalently attached cell-penetrating peptide: quantitative analysis of uptake and biological effect. Nucleic Acids Res..

[B56-pharmaceuticals-03-00961] Endoh T., Sisido M., Ohtsuki T. (2008). Cellular siRNA delivery mediated by a cell-permeant RNA-binding protein and photoinduced RNA interference. Bioconjug. Chem..

[B57-pharmaceuticals-03-00961] Mano M., Teodosio C., Paiva A., Simoes S., Pedroso de Lima M.C. (2005). On the mechanisms of the internalization of S4(13)-PV cell-penetrating peptide. Biochem. J..

[B58-pharmaceuticals-03-00961] Mueller J., Kretzschmar I., Volkmer R., Boisguerin P. (2008). Comparison of cellular uptake using 22 CPPs in 4 different cell lines. Bioconjug. Chem..

[B59-pharmaceuticals-03-00961] Trabulo S., Mano M., Faneca H., Cardoso A.L., Duarte S., Henriques A., Paiva A., Gomes P., Simoes S., de Lima M.C. (2008). S4(13)-PV cell penetrating peptide and cationic liposomes act synergistically to mediate intracellular delivery of plasmid DNA. J. Gene Med..

[B60-pharmaceuticals-03-00961] Mano M., Henriques A., Paiva A., Prieto M., Gavilanes F., Simoes S., de Lima M.C. (2007). Interaction of S413-PV cell penetrating peptide with model membranes: relevance to peptide translocation across biological membranes. J. Pept. Sci..

[B61-pharmaceuticals-03-00961] Mano M., Henriques A., Paiva A., Prieto M., Gavilanes F., Simoes S., Pedroso de Lima M.C. (2006). Cellular uptake of S413-PV peptide occurs upon conformational changes induced by peptide-membrane interactions. Biochim. Biophys. Acta.

[B62-pharmaceuticals-03-00961] Magzoub M., Kilk K., Eriksson L.E., Langel U., Graslund A. (2001). Interaction and structure induction of cell-penetrating peptides in the presence of phospholipid vesicles. Biochim. Biophys. Acta.

[B63-pharmaceuticals-03-00961] Magzoub M., Eriksson L.E., Graslund A. (2003). Comparison of the interaction, positioning, structure induction and membrane perturbation of cell-penetrating peptides and non-translocating variants with phospholipid vesicles. Biophys. Chem..

[B64-pharmaceuticals-03-00961] Thoren P.E., Persson D., Esbjorner E.K., Goksor M., Lincoln P., Norden B. (2004). Membrane binding and translocation of cell-penetrating peptides. Biochemistry.

[B65-pharmaceuticals-03-00961] Deshayes S., Heitz A., Morris M.C., Charnet P., Divita G., Heitz F. (2004). Insight into the mechanism of internalization of the cell-penetrating carrier peptide Pep-1 through conformational analysis. Biochemistry.

[B66-pharmaceuticals-03-00961] Magzoub M., Eriksson L.E., Graslund A. (2002). Conformational states of the cell-penetrating peptide penetratin when interacting with phospholipid vesicles: effects of surface charge and peptide concentration. Biochim. Biophys. Acta.

[B67-pharmaceuticals-03-00961] Joanne P., Galanth C., Goasdoue N., Nicolas P., Sagan S., Lavielle S., Chassaing G., El Amri C., Alves I.D. (2009). Lipid reorganization induced by membrane-active peptides probed using differential scanning calorimetry. Biochim. Biophys. Acta.

[B68-pharmaceuticals-03-00961] Deshayes S., Gerbal-Chaloin S., Morris M.C., Aldrian-Herrada G., Charnet P., Divita G., Heitz F. (2004). On the mechanism of non-endosomial peptide-mediated cellular delivery of nucleic acids. Biochim. Biophys. Acta.

[B69-pharmaceuticals-03-00961] Ziegler A., Blatter X.L., Seelig A., Seelig J. (2003). Protein transduction domains of HIV-1 and SIV TAT interact with charged lipid vesicles. Binding mechanism and thermodynamic analysis. Biochemistry.

[B70-pharmaceuticals-03-00961] Opalinska J.B., Gewirtz A.M. (2002). Nucleic-acid therapeutics: basic principles and recent applications. Nat. Rev. Drug Discov..

[B71-pharmaceuticals-03-00961] Said Hassane F., Saleh A.F., Abes R., Gait M.J., Lebleu B. (2009). Cell penetrating peptides: overview and applications to the delivery of oligonucleotides. Cell. Mol. Life Sci..

[B72-pharmaceuticals-03-00961] Veldhoen S., Laufer S.D., Restle T. (2008). Recent developments in Peptide-based nucleic Acid delivery. Int. J. Mol. Sci..

[B73-pharmaceuticals-03-00961] Torchilin V.P. (2008). Cell penetrating peptide-modified pharmaceutical nanocarriers for intracellular drug and gene delivery. Biopolymers.

[B74-pharmaceuticals-03-00961] Temsamani J., Vidal P. (2004). The use of cell-penetrating peptides for drug delivery. Drug Discov. Today.

[B75-pharmaceuticals-03-00961] Kilk K., El-Andaloussi S., Jarver P., Meikas A., Valkna A., Bartfai T., Kogerman P., Metsis M., Langel U. (2005). Evaluation of transportan 10 in PEI mediated plasmid delivery assay. J. Control. Release.

[B76-pharmaceuticals-03-00961] Rudolph C., Plank C., Lausier J., Schillinger U., Muller R.H., Rosenecker J. (2003). Oligomers of the arginine-rich motif of the HIV-1 TAT protein are capable of transferring plasmid DNA into cells. J. Biol. Chem..

[B77-pharmaceuticals-03-00961] Ignatovich I.A., Dizhe E.B., Pavlotskaya A.V., Akifiev B.N., Burov S.V., Orlov S.V., Perevozchikov A.P. (2003). Complexes of plasmid DNA with basic domain 47-57 of the HIV-1 Tat protein are transferred to mammalian cells by endocytosis-mediated pathways. J. Biol. Chem..

[B78-pharmaceuticals-03-00961] Kogure K., Moriguchi R., Sasaki K., Ueno M., Futaki S., Harashima H. (2004). Development of a non-viral multifunctional envelope-type nano device by a novel lipid film hydration method. J. Control. Release.

[B79-pharmaceuticals-03-00961] Kogure K., Akita H., Harashima H. (2007). Multifunctional envelope-type nano device for non-viral gene delivery: concept and application of Programmed Packaging. J. Control. Release.

[B80-pharmaceuticals-03-00961] Khalil I.A., Kogure K., Futaki S., Hama S., Akita H., Ueno M., Kishida H., Kudoh M., Mishina Y., Kataoka K., Yamada M., Harashima H. (2007). Octaarginine-modified multifunctional envelope-type nanoparticles for gene delivery. Gene Ther..

[B81-pharmaceuticals-03-00961] Tung C.H., Mueller S., Weissleder R. (2002). Novel branching membrane translocational peptide as gene delivery vector. Bioorg. Med. Chem..

[B82-pharmaceuticals-03-00961] Vandenbroucke R.E., De Smedt S.C., Demeester J., Sanders N.N. (2007). Cellular entry pathway and gene transfer capacity of TAT-modified lipoplexes. Biochim. Biophys. Acta.

[B83-pharmaceuticals-03-00961] Zhang C., Tang N., Liu X., Liang W., Xu W., Torchilin V.P. (2006). siRNA-containing liposomes modified with polyarginine effectively silence the targeted gene. J. Control. Release.

[B84-pharmaceuticals-03-00961] Kumar P., Wu H., McBride J.L., Jung K.E., Kim M.H., Davidson B.L., Lee S.K., Shankar P., Manjunath N. (2007). Transvascular delivery of small interfering RNA to the central nervous system. Nature.

[B85-pharmaceuticals-03-00961] Tan M.L., Choong P.F., Dass C.R. (2009). Recent developments in liposomes, microparticles and nanoparticles for protein and peptide drug delivery. Peptides.

[B86-pharmaceuticals-03-00961] Wadia J.S., Dowdy S.F. (2005). Transmembrane delivery of protein and peptide drugs by TAT-mediated transduction in the treatment of cancer. Adv. Drug Deliv. Rev..

[B87-pharmaceuticals-03-00961] Mi Z., Mai J., Lu X., Robbins P.D. (2000). Characterization of a class of cationic peptides able to facilitate efficient protein transduction *in vitro* and in vivo. Mol. Ther..

[B88-pharmaceuticals-03-00961] Fawell S., Seery J., Daikh Y., Moore C., Chen L.L., Pepinsky B., Barsoum J. (1994). Tat-mediated delivery of heterologous proteins into cells. Proc. Natl. Acad. Sci. USA.

[B89-pharmaceuticals-03-00961] Schwarze S.R., Ho A., Vocero-Akbani A., Dowdy S.F. (1999). In vivo protein transduction: delivery of a biologically active protein into the mouse. Science.

[B90-pharmaceuticals-03-00961] Caron N.J., Torrente Y., Camirand G., Bujold M., Chapdelaine P., Leriche K., Bresolin N., Tremblay J.P. (2001). Intracellular delivery of a Tat-eGFP fusion protein into muscle cells. Mol. Ther..

[B91-pharmaceuticals-03-00961] Cao G., Pei W., Ge H., Liang Q., Luo Y., Sharp F.R., Lu A., Ran R., Graham S.H., Chen J. (2002). In vivo Delivery of a Bcl-xL Fusion Protein Containing the TAT Protein Transduction Domain Protects against Ischemic Brain Injury and Neuronal Apoptosis. J. Neurosci..

[B92-pharmaceuticals-03-00961] Embury J., Klein D., Pileggi A., Ribeiro M., Jayaraman S., Molano R.D., Fraker C., Kenyon N., Ricordi C., Inverardi L., Pastori R.L. (2001). Proteins linked to a protein transduction domain efficiently transduce pancreatic islets. Diabetes.

[B93-pharmaceuticals-03-00961] Jin L.H., Bahn J.H., Eum W.S., Kwon H.Y., Jang S.H., Han K.H., Kang T.C., Won M.H., Kang J.H., Cho S.W., Park J., Choi S.Y. (2001). Transduction of human catalase mediated by an HIV-1 TAT protein basic domain and arginine-rich peptides into mammalian cells. Free Radic. Biol. Med..

[B94-pharmaceuticals-03-00961] Yoon H.Y., Lee S.H., Cho S.W., Lee J.E., Yoon C.S., Park J., Kim T.U., Choi S.Y. (2002). TAT-mediated delivery of human glutamate dehydrogenase into PC12 cells. Neurochem. Int..

[B95-pharmaceuticals-03-00961] Kwon H.Y., Eum W.S., Jang H.W., Kang J.H., Ryu J., Ryong Lee B., Jin L.H., Park J., Choi S.Y. (2000). Transduction of Cu,Zn-superoxide dismutase mediated by an HIV-1 Tat protein basic domain into mammalian cells. FEBS Lett..

[B96-pharmaceuticals-03-00961] Kabouridis P.S., Hasan M., Newson J., Gilroy D.W., Lawrence T. (2002). Inhibition of NF-kappa B activity by a membrane-transducing mutant of I kappa B alpha. J. Immunol..

[B97-pharmaceuticals-03-00961] Wheeler D.S., Dunsmore K.E., Wong H.R. (2003). Intracellular delivery of HSP70 using HIV-1 Tat protein transduction domain. Biochem. Biophys. Res. Commun..

[B98-pharmaceuticals-03-00961] Deshayes S., Morris M., Heitz F., Divita G. (2008). Delivery of proteins and nucleic acids using a non-covalent peptide-based strategy. Adv. Drug Deliv. Rev..

[B99-pharmaceuticals-03-00961] Gros E., Deshayes S., Morris M.C., Aldrian-Herrada G., Depollier J., Heitz F., Divita G. (2006). A non-covalent peptide-based strategy for protein and peptide nucleic acid transduction. Biochim. Biophys. Acta.

[B100-pharmaceuticals-03-00961] Torchilin V.P. (2006). Multifunctional nanocarriers. Adv. Drug Deliv. Rev..

[B101-pharmaceuticals-03-00961] McCarthy J.R., Kelly K.A., Sun E.Y., Weissleder R. (2007). Targeted delivery of multifunctional magnetic nanoparticles. Nanomed..

[B102-pharmaceuticals-03-00961] McCarthy J.R., Weissleder R. (2008). Multifunctional magnetic nanoparticles for targeted imaging and therapy. Adv. Drug Deliv. Rev..

[B103-pharmaceuticals-03-00961] Josephson L., Tung C.H., Moore A., Weissleder R. (1999). High-efficiency intracellular magnetic labeling with novel superparamagnetic-Tat peptide conjugates. Bioconjug. Chem..

[B104-pharmaceuticals-03-00961] Lewin M., Carlesso N., Tung C.H., Tang X.W., Cory D., Scadden D.T., Weissleder R. (2000). Tat peptide-derivatized magnetic nanoparticles allow *in vivo* tracking and recovery of progenitor cells. Nat. Biotechnol..

[B105-pharmaceuticals-03-00961] Torchilin V.P., Levchenko T.S., Rammohan R., Volodina N., Papahadjopoulos-Sternberg B., D'Souza G.G. (2003). Cell transfection *in vitro* and *in vivo* with nontoxic TAT peptide-liposome-DNA complexes. Proc. Natl. Acad. Sci. U. S. A..

[B106-pharmaceuticals-03-00961] Tseng Y.L., Liu J.J., Hong R.L. (2002). Translocation of liposomes into cancer cells by cell-penetrating peptides penetratin and tat: a kinetic and efficacy study. Mol. Pharmacol..

[B107-pharmaceuticals-03-00961] Jarver P., Langel U. (2004). The use of cell-penetrating peptides as a tool for gene regulation. Drug Discov. Today.

[B108-pharmaceuticals-03-00961] Laufer S.D., Restle T. (2008). Peptide-mediated cellular delivery of oligonucleotide-based therapeutics *in vitro*: quantitative evaluation of overall efficacy employing easy to handle reporter systems. Curr. Pharm. Des..

[B109-pharmaceuticals-03-00961] Gleave M.E., Monia B.P. (2005). Antisense therapy for cancer. Nat. Rev. Cancer.

[B110-pharmaceuticals-03-00961] Juliano R., Bauman J., Kang H., Ming X. (2009). Biological barriers to therapy with antisense and siRNA oligonucleotides. Mol. Pharm..

[B111-pharmaceuticals-03-00961] Juliano R., Alam M.R., Dixit V., Kang H. (2008). Mechanisms and strategies for effective delivery of antisense and siRNA oligonucleotides. Nucleic Acids Res..

[B112-pharmaceuticals-03-00961] Kurreck J. (2003). Antisense technologies. Improvement through novel chemical modifications. Eur. J. Biochem..

[B113-pharmaceuticals-03-00961] Lebleu B., Moulton H.M., Abes R., Ivanova G.D., Abes S., Stein D.A., Iversen P.L., Arzumanov A.A., Gait M.J. (2008). Cell penetrating peptide conjugates of steric block oligonucleotides. Adv. Drug Deliv. Rev..

[B114-pharmaceuticals-03-00961] Gait M.J. (2003). Peptide-mediated cellular delivery of antisense oligonucleotides and their analogues. Cell. Mol. Life Sci..

[B115-pharmaceuticals-03-00961] Morris M.C., Vidal P., Chaloin L., Heitz F., Divita G. (1997). A new peptide vector for efficient delivery of oligonucleotides into mammalian cells. Nucleic Acids Res..

[B116-pharmaceuticals-03-00961] Zatsepin T.S., Turner J.J., Oretskaya T.S., Gait M.J. (2005). Conjugates of oligonucleotides and analogues with cell penetrating peptides as gene silencing agents. Curr. Pharm. Des..

[B117-pharmaceuticals-03-00961] Pooga M., Soomets U., Hallbrink M., Valkna A., Saar K., Rezaei K., Kahl U., Hao J.X., Xu X.J., Wiesenfeld-Hallin Z., Hokfelt T., Bartfai T., Langel U. (1998). Cell penetrating PNA constructs regulate galanin receptor levels and modify pain transmission *in vivo*. Nat. Biotechnol..

[B118-pharmaceuticals-03-00961] Abes S., Moulton H.M., Clair P., Prevot P., Youngblood D.S., Wu R.P., Iversen P.L., Lebleu B. (2006). Vectorization of morpholino oligomers by the (R-Ahx-R)4 peptide allows efficient splicing correction in the absence of endosomolytic agents. J. Control. Release.

[B119-pharmaceuticals-03-00961] Abes R., Moulton H.M., Clair P., Yang S.T., Abes S., Melikov K., Prevot P., Youngblood D.S., Iversen P.L., Chernomordik L.V., Lebleu B. (2008). Delivery of steric block morpholino oligomers by (R-X-R)4 peptides: structure-activity studies. Nucleic Acids Res..

[B120-pharmaceuticals-03-00961] Laufer S.D., Recke A.L., Veldhoen S., Trampe A., Restle T. (2009). Noncovalent Peptide-Mediated Delivery of Chemically Modified Steric Block Oligonucleotides Promotes Splice Correction: Quantitative Analysis of Uptake and Biological Effect. Oligonucleotides.

[B121-pharmaceuticals-03-00961] Lehto T., Abes R., Oskolkov N., Suhorutsenko J., Copolovici D.M., Mager I., Viola J.R., Simonson O.E., Ezzat K., Guterstam P., Eriste E., Smith C.I., Lebleu B., Samir El A., Langel U. (2009). Delivery of nucleic acids with a stearylated (RxR)(4) peptide using a non-covalent co-incubation strategy. J. Control. Release.

[B122-pharmaceuticals-03-00961] Mae M., El Andaloussi S., Lundin P., Oskolkov N., Johansson H.J., Guterstam P., Langel U. (2009). A stearylated CPP for delivery of splice correcting oligonucleotides using a non-covalent co-incubation strategy. J. Control. Release.

[B123-pharmaceuticals-03-00961] Morris M.C., Chaloin L., Choob M., Archdeacon J., Heitz F., Divita G. (2004). Combination of a new generation of PNAs with a peptide-based carrier enables efficient targeting of cell cycle progression. Gene Ther..

[B124-pharmaceuticals-03-00961] Morris M.C., Gros E., Aldrian-Herrada G., Choob M., Archdeacon J., Heitz F., Divita G. (2007). A non-covalent peptide-based carrier for *in vivo *delivery of DNA mimics. Nucleic Acids Res..

[B125-pharmaceuticals-03-00961] Oehlke J., Wallukat G., Wolf Y., Ehrlich A., Wiesner B., Berger H., Bienert M. (2004). Enhancement of intracellular concentration and biological activity of PNA after conjugation with a cell-penetrating synthetic model peptide. Eur. J. Biochem..

[B126-pharmaceuticals-03-00961] Kang S.H., Cho M.J., Kole R. (1998). Up-regulation of luciferase gene expression with antisense oligonucleotides: implications and applications in functional assay development. Biochemistry.

[B127-pharmaceuticals-03-00961] Turner J.J., Jones S., Fabani M.M., Ivanova G., Arzumanov A.A., Gait M.J. (2007). RNA targeting with peptide conjugates of oligonucleotides, siRNA and PNA. Blood Cells Mol. Dis..

[B128-pharmaceuticals-03-00961] Moulton H.M., Nelson M.H., Hatlevig S.A., Reddy M.T., Iversen P.L. (2004). Cellular uptake of antisense morpholino oligomers conjugated to arginine-rich peptides. Bioconjug. Chem..

[B129-pharmaceuticals-03-00961] Abes S., Williams D., Prevot P., Thierry A., Gait M.J., Lebleu B. (2006). Endosome trapping limits the efficiency of splicing correction by PNA-oligolysine conjugates. J. Control. Release.

[B130-pharmaceuticals-03-00961] Shiraishi T., Pankratova S., Nielsen P.E. (2005). Calcium ions effectively enhance the effect of antisense peptide nucleic acids conjugated to cationic tat and oligoarginine peptides. Chem. Biol..

[B131-pharmaceuticals-03-00961] Wolf Y., Pritz S., Abes S., Bienert M., Lebleu B., Oehlke J. (2006). Structural requirements for cellular uptake and antisense activity of peptide nucleic acids conjugated with various peptides. Biochemistry.

[B132-pharmaceuticals-03-00961] Abes S., Moulton H., Turner J., Clair P., Richard J.P., Iversen P., Gait M.J., Lebleu B. (2007). Peptide-based delivery of nucleic acids: design, mechanism of uptake and applications to splice-correcting oligonucleotides. Biochem. Soc. Trans..

[B133-pharmaceuticals-03-00961] El-Andaloussi S., Johansson H.J., Lundberg P., Langel U. (2006). Induction of splice correction by cell-penetrating peptide nucleic acids. J. Gene Med..

[B134-pharmaceuticals-03-00961] Berg K., Prasmickaite L., Selbo P.K., Hellum M., Bonsted A., Hogset A. (2003). Photochemical internalization (PCI)--a novel technology for release of macromolecules from endocytic vesicles. Oftalmologia.

[B135-pharmaceuticals-03-00961] Boe S., Hovig E. (2006). Photochemically induced gene silencing using PNA-peptide conjugates. Oligonucleotides.

[B136-pharmaceuticals-03-00961] Shiraishi T., Bendifallah N., Nielsen P.E. (2006). Cellular delivery of polyheteroaromate-peptide nucleic acid conjugates mediated by cationic lipids. Bioconjug. Chem..

[B137-pharmaceuticals-03-00961] Shiraishi T., Nielsen P.E. (2006). Photochemically enhanced cellular delivery of cell penetrating peptide-PNA conjugates. FEBS Lett..

[B138-pharmaceuticals-03-00961] Abes S., Turner J.J., Ivanova G.D., Owen D., Williams D., Arzumanov A., Clair P., Gait M.J., Lebleu B. (2007). Efficient splicing correction by PNA conjugation to an R6-Penetratin delivery peptide. Nucleic Acids Res..

[B139-pharmaceuticals-03-00961] Jearawiriyapaisarn N., Moulton H.M., Buckley B., Roberts J., Sazani P., Fucharoen S., Iversen P.L., Kole R. (2008). Sustained dystrophin expression induced by peptide-conjugated morpholino oligomers in the muscles of mdx mice. Mol. Ther..

[B140-pharmaceuticals-03-00961] Yin H., Moulton H.M., Betts C., Seow Y., Boutilier J., Iverson P.L., Wood M.J. (2009). A fusion peptide directs enhanced systemic dystrophin exon skipping and functional restoration in dystrophin-deficient mdx mice. Hum. Mol. Genet..

[B141-pharmaceuticals-03-00961] Yin H., Moulton H.M., Seow Y., Boyd C., Boutilier J., Iverson P., Wood M.J. (2008). Cell-penetrating peptide-conjugated antisense oligonucleotides restore systemic muscle and cardiac dystrophin expression and function. Hum. Mol. Genet..

[B142-pharmaceuticals-03-00961] Trabulo S., Resina S., Simões S., Lebleu B., Pedroso de Lima M.C. (2010). A non-covalent strategy combining cationic lipids and CPPs to enhance the delivery of splice correcting oligonucleotides. J. Control. Release.

[B143-pharmaceuticals-03-00961] Grunweller A., Hartmann R.K. (2005). RNA interference as a gene-specific approach for molecular medicine. Curr. Med. Chem..

[B144-pharmaceuticals-03-00961] Lu P.Y., Xie F., Woodle M.C. (2005). In vivo application of RNA interference: from functional genomics to therapeutics. Adv. Genet..

[B145-pharmaceuticals-03-00961] Hannon G.J. (2002). RNA interference. Nature.

[B146-pharmaceuticals-03-00961] Dorsett Y., Tuschl T. (2004). siRNAs: applications in functional genomics and potential as therapeutics. Nat. Rev. Drug Discov..

[B147-pharmaceuticals-03-00961] de Fougerolles A., Vornlocher H.P., Maraganore J., Lieberman J. (2007). Interfering with disease: a progress report on siRNA-based therapeutics. Nat. Rev. Drug Discov..

[B148-pharmaceuticals-03-00961] Eguchi A., Dowdy S.F. (2009). siRNA delivery using peptide transduction domains. Trends Pharmacol. Sci..

[B149-pharmaceuticals-03-00961] Endoh T., Ohtsuki T. (2009). Cellular siRNA delivery using cell-penetrating peptides modified for endosomal escape. Adv. Drug Deliv. Rev..

[B150-pharmaceuticals-03-00961] Muratovska A., Eccles M.R. (2004). Conjugate for efficient delivery of short interfering RNA (siRNA) into mammalian cells. FEBS Lett..

[B151-pharmaceuticals-03-00961] Chiu Y.L., Ali A., Chu C.Y., Cao H., Rana T.M. (2004). Visualizing a correlation between siRNA localization, cellular uptake, and RNAi in living cells. Chem. Biol..

[B152-pharmaceuticals-03-00961] Davidson T.J., Harel S., Arboleda V.A., Prunell G.F., Shelanski M.L., Greene L.A., Troy C.M. (2004). Highly efficient small interfering RNA delivery to primary mammalian neurons induces MicroRNA-like effects before mRNA degradation. J. Neurosci..

[B153-pharmaceuticals-03-00961] Meade B.R., Dowdy S.F. (2008). Enhancing the cellular uptake of siRNA duplexes following noncovalent packaging with protein transduction domain peptides. Adv. Drug Deliv. Rev..

[B154-pharmaceuticals-03-00961] Turner J.J., Ivanova G.D., Verbeure B., Williams D., Arzumanov A.A., Abes S., Lebleu B., Gait M.J. (2005). Cell-penetrating peptide conjugates of peptide nucleic acids (PNA) as inhibitors of HIV-1 Tat-dependent trans-activation in cells. Nucleic Acids Res..

[B155-pharmaceuticals-03-00961] Simeoni F., Morris M.C., Heitz F., Divita G. (2003). Insight into the mechanism of the peptide-based gene delivery system MPG: implications for delivery of siRNA into mammalian cells. Nucleic Acids Res..

[B156-pharmaceuticals-03-00961] Lundberg P., El-Andaloussi S., Sutlu T., Johansson H., Langel U. (2007). Delivery of short interfering RNA using endosomolytic cell-penetrating peptides. FASEB J..

[B157-pharmaceuticals-03-00961] Crombez L., Charnet A., Morris M.C., Aldrian-Herrada G., Heitz F., Divita G. (2007). A non-covalent peptide-based strategy for siRNA delivery. Biochem. Soc. Trans..

[B158-pharmaceuticals-03-00961] Eguchi A., Meade B.R., Chang Y.C., Fredrickson C.T., Willert K., Puri N., Dowdy S.F. (2009). Efficient siRNA delivery into primary cells by a peptide transduction domain-dsRNA binding domain fusion protein. Nat. Biotechnol..

[B159-pharmaceuticals-03-00961] Kim W.J., Christensen L.V., Jo S., Yockman J.W., Jeong J.H., Kim Y.H., Kim S.W. (2006). Cholesteryl oligoarginine delivering vascular endothelial growth factor siRNA effectively inhibits tumor growth in colon adenocarcinoma. Mol. Ther..

[B160-pharmaceuticals-03-00961] Zeineddine D., Papadimou E., Chebli K., Gineste M., Liu J., Grey C., Thurig S., Behfar A., Wallace V.A., Skerjanc I.S., Puceat M. (2006). Oct-3/4 dose dependently regulates specification of embryonic stem cells toward a cardiac lineage and early heart development. Dev. Cell.

[B161-pharmaceuticals-03-00961] Crombez L., Morris M.C., Dufort S., Aldrian-Herrada G., Nguyen Q., Mc Master G., Coll J.L., Heitz F., Divita G. (2009). Targeting cyclin B1 through peptide-based delivery of siRNA prevents tumour growth. Nucleic Acids Res..

[B162-pharmaceuticals-03-00961] Li S., H. L. (1996). Lipidic supramolecular Assemblies for Gene Transfer. J. Liposome Res..

[B163-pharmaceuticals-03-00961] Simoes S., Slepushkin V., Pires P., Gaspar R., de Lima M.P., Duzgunes N. (1999). Mechanisms of gene transfer mediated by lipoplexes associated with targeting ligands or pH-sensitive peptides. Gene Ther..

[B164-pharmaceuticals-03-00961] de Fougerolles A.R. (2008). Delivery vehicles for small interfering RNA *in vivo*. Hum. Gene Ther..

[B165-pharmaceuticals-03-00961] Glover D.J., Lipps H.J., Jans D.A. (2005). Towards safe, non-viral therapeutic gene expression in humans. Nat. Rev. Genet..

[B166-pharmaceuticals-03-00961] Simoes S., Slepushkin V., Gaspar R., de Lima M.C., Duzgunes N. (1998). Gene delivery by negatively charged ternary complexes of DNA, cationic liposomes and transferrin or fusigenic peptides. Gene Ther..

[B167-pharmaceuticals-03-00961] Li S., Huang L. (2000). Nonviral gene therapy: promises and challenges. Gene Ther..

[B168-pharmaceuticals-03-00961] Martin M.E., Rice K.G. (2007). Peptide-guided gene delivery. AAPS J..

[B169-pharmaceuticals-03-00961] Ogris M., Wagner E. (2002). Targeting tumors with non-viral gene delivery systems. Drug Discov. Today.

[B170-pharmaceuticals-03-00961] Morris M.C., Chaloin L., Mery J., Heitz F., Divita G. (1999). A novel potent strategy for gene delivery using a single peptide vector as a carrier. Nucleic Acids Res..

[B171-pharmaceuticals-03-00961] Rittner K., Benavente A., Bompard-Sorlet A., Heitz F., Divita G., Brasseur R., Jacobs E. (2002). New basic membrane-destabilizing peptides for plasmid-based gene delivery *in vitro* and *in vivo*. Mol. Ther..

[B172-pharmaceuticals-03-00961] Futaki S., Ohashi W., Suzuki T., Niwa M., Tanaka S., Ueda K., Harashima H., Sugiura Y. (2001). Stearylated arginine-rich peptides: a new class of transfection systems. Bioconjug. Chem..

[B173-pharmaceuticals-03-00961] Khalil I.A., Futaki S., Niwa M., Baba Y., Kaji N., Kamiya H., Harashima H. (2004). Mechanism of improved gene transfer by the N-terminal stearylation of octaarginine: enhanced cellular association by hydrophobic core formation. Gene Ther..

[B174-pharmaceuticals-03-00961] Sandgren S., Cheng F., Belting M. (2002). Nuclear targeting of macromolecular polyanions by an HIV-Tat derived peptide. Role for cell-surface proteoglycans. J. Biol. Chem..

[B175-pharmaceuticals-03-00961] Lo S.L., Wang S. (2008). An endosomolytic Tat peptide produced by incorporation of histidine and cysteine residues as a nonviral vector for DNA transfection. Biomaterials.

[B176-pharmaceuticals-03-00961] Torchilin V.P. (2008). Tat peptide-mediated intracellular delivery of pharmaceutical nanocarriers. Adv. Drug Deliv. Rev..

[B177-pharmaceuticals-03-00961] Vives E., Schmidt J., Pelegrin A. (2008). Cell-penetrating and cell-targeting peptides in drug delivery. Biochim. Biophys. Acta.

[B178-pharmaceuticals-03-00961] Branden L.J., Mohamed A.J., Smith C.I. (1999). A peptide nucleic acid-nuclear localization signal fusion that mediates nuclear transport of DNA. Nat. Biotechnol..

[B179-pharmaceuticals-03-00961] Kleemann E., Neu M., Jekel N., Fink L., Schmehl T., Gessler T., Seeger W., Kissel T. (2005). Nano-carriers for DNA delivery to the lung based upon a TAT-derived peptide covalently coupled to PEG-PEI. J. Control. Release.

[B180-pharmaceuticals-03-00961] Torchilin V.P., Rammohan R., Weissig V., Levchenko T.S. (2001). TAT peptide on the surface of liposomes affords their efficient intracellular delivery even at low temperature and in the presence of metabolic inhibitors. Proc. Natl. Acad. Sci. USA.

[B181-pharmaceuticals-03-00961] MacKay J.A., Li W., Huang Z., Dy E.E., Huynh G., Tihan T., Collins R., Deen D.F., Szoka Jr. F.C. (2008). HIV TAT peptide modifies the distribution of DNA nanolipoparticles following convection-enhanced delivery. Mol. Ther..

[B182-pharmaceuticals-03-00961] Hyndman L., Lemoine J.L., Huang L., Porteous D.J., Boyd A.C., Nan X. (2004). HIV-1 Tat protein transduction domain peptide facilitates gene transfer in combination with cationic liposomes. J. Control. Release.

[B183-pharmaceuticals-03-00961] El-Sayed A., Futaki S., Harashima H. (2009). Delivery of macromolecules using arginine-rich cell-penetrating peptides: ways to overcome endosomal entrapment. AAPS J..

[B184-pharmaceuticals-03-00961] El-Sayed A., Khalil I.A., Kogure K., Futaki S., Harashima H. (2008). Octaarginine- and octalysine-modified nanoparticles have different modes of endosomal escape. J. Biol. Chem..

